# Supplementary material for: Accounting for multiple imputation-induced variability for differential analysis in mass spectrometry-based label-free quantitative proteomics
Source: PLoS Comput Biol. 2022 Aug 29;18(8):e1010420. doi: 10.1371/journal.pcbi.1010420 (PMC9462777; doi:10.1371/journal.pcbi.1010420)
Supplement: S1 Table — This table gives an overview of the recent literature on imputation methods in quantitative proteomics. (PDF) [file pcbi.1010420.s001.pdf]

S1 Table. State of the art on imputation in quantitative proteomics. This table gives an overview of the recent literature on imputation methods in quantitative proteomics. Imputation methods are abbreviated as follows.

BPCA: Bayesian principal component analysis

CAM: Convex analysis of mixtures

FCS: Fully conditional specification

FRMF: Fused regularisation matrix factorisation

kNN: k-nearest neighbours

LLS: Local least-squares

LOD1: Half of the global minimum

LOD2: Half of the peptide minimum

LSA: Least-squares adaptive

MBI: Model-based imputation

MCMC: Monte-Carlo Markov chains

MI: Multiple imputation

mice: Multiple imputation using chained equations

MinDet: Deterministic minimum

MinProb: Probabilistic minimum

MLE: Maximum likelihood estimation

NIPALS: Non-linear estimation by iterative partial least squares

PCA: Principal component analysis

PPCA: Probabilistic principal component analysis

pwKNN: Protein-wise k-nearest neighbours

QRLIC: Quantile regression imputation of left-censored

SLSA: Structured least squares algorithm

SVD: Singular value decomposition

SVT: Singular value thresholding

swKNN: Sample-wise k-nearest neighbours

REM: Regularised expectation maximisation

RF: Random forests

RTI: Random tail imputation

References	Imputation methods	Evaluation datasets
[1]	**Single imputation:** MLE	**Simulated dataset:**<br>10 samples, 2 groups, 1400 proteins
[2]	**Single imputation:** Accelerated Failure Time model	
[3]	**Single imputation:**<br>Single-Value Approaches (LOD1, LOD2, RTI)<br>Local Similarity Approaches (KNN, LLS, LSA, REM, MBI)<br>Global-Structure Approaches (PPCA and BPCA)	**Real datasets:**<br>Mouse plasma + Shewanella oneidensis, 60 samples, 1518 peptides<br>Human Plasma, 71 samples, 48 vs 23 T2D, 6729 peptides<br>Mouse Lung, 32 samples, 6295 peptides
[4]	**Single imputation:** kNN, SVD, MLE, MinDet, MinProb	**Simulated dataset:** [1]<br>1000 peptides, 20 replicates<br>**Real dataset:** [5]
[6]	**Multiple imputation:** MCMC + FCS	**Real dataset:**<br>Framingham Heart Study Offspring cohort<br>861 plasma proteins, 135 samples<br>MCAR amputation on the 261 entirely observed proteins<br>Application to 544 partially unobserved proteins (40% missing values)
[7]	**Single imputation:**<br>Two-step lasso method, kNN, TR-kNN, RF, DanteR, Min	**Real datasets:**<br>[8, 9, 10]
[11]	Hurdle model	**Real dataset:** [12]
[13]	**Multiple imputation:**<br>MI, PCA, MLE, kNN, IGCDA, RF, SLSA	**Simulated dataset:** [14]
[15]	**Single imputation:**<br>BPCA, kNN, MinProb, MLE, QRLIC, SVD, DetMin	**Real datasets:** 1-4 groups, 9-56 samples, 1847-6932 proteins<br>Available on PRIDE repositories<br><br>**Simulated datasets:** Based on the real datasets<br>3 groups, 27-60 samples, 2800-3500 proteins
[16]	**Single imputation:**<br>left-censored methods, kNN, LLS, RF, SVD, BPCA	**Real datasets:**<br>(E.coli + Yeast) + UPS, 7 groups, 56 samples<br>Immune cell dataset, 3 vs 4 samples<br>Amputation of complete cases
[17]	**Single imputation:**<br>swKNN, pwKNN, Min/2, Mean, PPCA, NIPALS, SVD,<br>SVT, FRMF, CAM	**Real dataset:**<br>[18]<br>Amputation of complete cases from real datasets
[19]	**Single imputation:** XGboost, mean, kNN, BPCA, LLS, RF	**Real datasets:**<br>Kinases expression of human colon<br>and rectal cancer cell line : 65 samples, 235 kinases<br>Proteome about the interstitial lung disease : 11 samples,<br>random draw of 500 completely observed proteins<br>Ovarian cancer proteome dataset : 25 samples,<br>random draw of 400 completely observed proteins

References

[1] Karpievitch YV, Dabney AR, Smith RD. Normalization and Missing Value Imputation for Label-Free LC-MS Analysis. BMC Bioinformatics. 2012;13(16):S5. doi:10.1186/1471-2105-13-S16-S5.

[2] Choi M, Chang CY, Clough T, Broudy D, Killeen T, MacLean B, et al. MSstats: An R Package for Statistical Analysis of Quantitative Mass Spectrometry-Based Proteomic Experiments. Bioinformatics. 2014;30(17):2524–2526. doi:10.1093/bioinformatics/btu305.

[3] Webb-Robertson BJM, Wiberg HK, Matzke MM, Brown JN, Wang J, McDermott JE, et al. Review, Evaluation, and Discussion of the Challenges of Missing Value Imputation for Mass Spectrometry-Based Label-Free Global Proteomics. Journal of Proteome Research. 2015;14(5):1993–2001. doi:10.1021/pr501138h.

[4] Lazar C, Gatto L, Ferro M, Bruley C, Burger T. Accounting for the Multiple Natures of Missing Values in Label-Free Quantitative Proteomics Data Sets to Compare Imputation Strategies. Journal of Proteome Research. 2016;15(4):1116–1125. doi:10.1021/acs.jproteome.5b00981.

[5] Zhang W, Wei Y, Ignatchenko V, Li L, Sakashita S, Pham NA, et al. Proteomic Profiles of Human Lung Adeno and Squamous Cell Carcinoma Using Super-SILAC and Label-Free Quantification Approaches. PROTEOMICS. 2014;14(6):795–803. doi:10.1002/pmic.201300382.

[6] Yin X, Levy D, Willinger C, Adourian A, Larson MG. Multiple Imputation and Analysis for High-Dimensional Incomplete Proteomics Data. Statistics in Medicine. 2016;35(8):1315–1326. doi:10.1002/sim.6800.

[7] Li Q, Fisher K, Meng W, Fang B, Welsh E, Haura EB, et al. GMSimpute: A Generalized Two-Step Lasso Approach to Impute Missing Values in Label-Free Mass Spectrum Analysis. Bioinformatics. 2020;36(1):257–263. doi:10.1093/bioinformatics/btz488.

[8] Bai Y, Kim JY, Watters JM, Fang B, Kinose F, Song L, et al. Adaptive Responses to Dasatinib-Treated Lung Squamous Cell Cancer Cells Harboring DDR2 Mutations. Cancer Research. 2014;74(24):7217–7228. doi:10.1158/0008-5472.CAN-14-0505.

[9] Kirwan JA, Weber RJM, Broadhurst DI, Viant MR. Direct Infusion Mass Spectrometry Metabolomics Dataset: A Benchmark for Data Processing and Quality Control. Scientific Data. 2014;1(1):140012. doi:10.1038/sdata.2014.12.

[10] Fang B, Hoffman MA, Mirza AS, Mishall KM, Li J, Peterman SM, et al. Evaluating Kinase ATP Uptake and Tyrosine Phosphorylation Using Multiplexed Quantification of Chemically Labeled and

Post-Translationally Modified Peptides. Methods (San Diego, Calif). 2015;81:41–49. doi:10.1016/j.ymeth.2015.03.006.

[11] Goeminne LJE, Sticker A, Martens L, Gevaert K, Clement L. MSqRob Takes the Missing Hurdle: Uniting Intensity- and Count-Based Proteomics. Analytical Chemistry. 2020;92(9):6278–6287. doi:10.1021/acs.analchem.9b04375.

[12] Paulovich AG, Billheimer D, Ham AJL, Vega-Montoto L, Rudnick PA, Tabb DL, et al. Interlaboratory Study Characterizing a Yeast Performance Standard for Benchmarking LC-MS Platform Performance *. Molecular & Cellular Proteomics. 2010;9(2):242–254. doi:10.1074/mcp.M900222-MCP200.

[13] Giai Gianetto Q, Wieczorek S, Couté Y, Burger T. A Peptide-Level Multiple Imputation Strategy Accounting for the Different Natures of Missing Values in Proteomics Data. bioRxiv. 2020; p. 2020.05.29.122770. doi:10.1101/2020.05.29.122770.

[14] Ramus C, Hovasse A, Marcellin M, Hesse AM, Mouton-Barbosa E, Bouyssié D, et al. Benchmarking Quantitative Label-Free LC-MS Data Processing Workflows Using a Complex Spiked Proteomic Standard Dataset. Journal of Proteomics. 2016;132:51–62. doi:10.1016/j.jprot.2015.11.011.

[15] Liu M, Dongre A. Proper Imputation of Missing Values in Proteomics Datasets for Differential Expression Analysis. Briefings in Bioinformatics. 2020;doi:10.1093/bib/bbaa112.

[16] Jin L, Bi Y, Hu C, Qu J, Shen S, Wang X, et al. A Comparative Study of Evaluating Missing Value Imputation Methods in Label-Free Proteomics. Scientific Reports. 2021;11(1):1760. doi:10.1038/s41598-021-81279-4.

[17] Shen M, Chang YT, Wu CT, Parker SJ, Saylor G, Wang Y, et al. Comparative Assessment and Outlook on Methods for Imputing Proteomics Data. In Review; 2021.

[18] Herrington DM, Mao C, Parker SJ, Fu Z, Yu G, Chen L, et al. Proteomic Architecture of Human Coronary and Aortic Atherosclerosis. Circulation. 2018;137(25):2741–2756. doi:10.1161/CIRCULATIONAHA.118.034365.

[19] Song J, Yu C. Missing Value Imputation Using XGboost for Label-Free Mass Spectrometry-Based Proteomics Data. Bioinformatics; 2021.
