# Supplementary material for: Accounting for multiple imputation-induced variability for differential analysis in mass spectrometry-based label-free quantitative proteomics
Source: PLoS Comput Biol. 2022 Aug 29;18(8):e1010420. doi: 10.1371/journal.pcbi.1010420 (PMC9462777; doi:10.1371/journal.pcbi.1010420)
Supplement: S2 Table — Results are provided as mean ± standard deviation over the 100 simulated datasets for each indicator of performance. (PDF) [file pcbi.1010420.s002.pdf]

%MV	Method	True positives	False positives	True negatives	False negatives	Sensitivity (%)	Specificity (%)	Precision (%)	F-score (%)	MCC (%)
**1%**	**DAPAR**	10 ± 0	0.5 ± 0.7	189.5 ± 0.7	0 ± 0	100 ± 0	99.8 ± 0.4	95.9 ± 5.7	97.8 ± 3.1	97.8 ± 3.1
	**MI4P**	10 ± 0	0.5 ± 0.7	189.5 ± 0.7	0 ± 0	100 ± 0	99.8 ± 0.4	96 ± 5.7	97.9 ± 3.1	97.8 ± 3.1
**5%**	**DAPAR**	10 ± 0	0.8 ± 1	189.2 ± 1	0 ± 0	100 ± 0	99.6 ± 0.5	92.9 ± 7.6	96.2 ± 4.2	96.1 ± 4.2
	**MI4P**	10 ± 0	0.5 ± 0.7	189.5 ± 0.7	0 ± 0	100 ± 0	99.8 ± 0.4	95.9 ± 6.1	97.8 ± 3.3	97.8 ± 3.4
**10%**	**DAPAR**	10 ± 0	1.2 ± 1.3	188.8 ± 1.3	0 ± 0	100 ± 0	99.4 ± 0.7	90.3 ± 9.3	94.6 ± 5.4	94.6 ± 5.3
	**MI4P**	10 ± 0	0.6 ± 0.8	189.4 ± 0.8	0 ± 0	100 ± 0	99.7 ± 0.4	95.3 ± 6.8	97.5 ± 3.7	97.4 ± 3.8
**15%**	**DAPAR**	10 ± 0	1.3 ± 1.3	188.7 ± 1.3	0 ± 0	100 ± 0	99.3 ± 0.7	89.6 ± 9.4	94.2 ± 5.4	94.2 ± 5.4
	**MI4P**	10 ± 0	0.6 ± 1	189.4 ± 1	0 ± 0	100 ± 0	99.7 ± 0.5	95.3 ± 7.4	97.4 ± 4.2	97.4 ± 4.2
**20%**	**DAPAR**	10 ± 0	2.2 ± 1.7	187.7 ± 1.7	0 ± 0	100 ± 0	98.8 ± 0.9	83.1 ± 10.9	90.4 ± 6.6	90.5 ± 6.4
	**MI4P**	10 ± 0	1.3 ± 1.7	188.6 ± 1.8	0 ± 0	100 ± 0	99.3 ± 0.9	89.8 ± 11.4	94.2 ± 6.7	94.3 ± 6.6
**25%**	**DAPAR**	10 ± 0.2	2.9 ± 2.1	186.8 ± 2.2	0 ± 0	100 ± 0	98.5 ± 1.1	79.7 ± 12.5	88.2 ± 7.9	88.3 ± 7.5
	**MI4P**	10 ± 0.2	1.6 ± 1.8	188 ± 2.1	0 ± 0	100 ± 0	99.2 ± 1	88.3 ± 12	93.3 ± 7.2	93.4 ± 7

S2 Table. Performance evaluation on the first set of MAR simulations imputed using maximum likelihood estimation. Results are provided as mean ± standard deviation over the 100 simulated datasets for each indicator of performance.
