# Supplementary material for: Accounting for multiple imputation-induced variability for differential analysis in mass spectrometry-based label-free quantitative proteomics
Source: PLoS Comput Biol. 2022 Aug 29;18(8):e1010420. doi: 10.1371/journal.pcbi.1010420 (PMC9462777; doi:10.1371/journal.pcbi.1010420)
Supplement: S3 Table — Results are provided as mean ± standard deviation over the 100 simulated datasets for each indicator of performance. (PDF) [file pcbi.1010420.s003.pdf]

%MV	Method	True positives	False positives	True negatives	False negatives	Sensitivity (%)	Specificity (%)	Precision (%)	F-score (%)	MCC (%)
**1%**	**DAPAR**	10 ± 0	0.4 ± 0.6	189.6 ± 0.6	0 ± 0	100 ± 0	99.8 ± 0.3	96.3 ± 5.4	98 ± 2.9	98 ± 2.9
	**MI4P**	10 ± 0	0.4 ± 0.6	189.6 ± 0.6	0 ± 0	100 ± 0	99.8 ± 0.3	96.3 ± 5.4	98 ± 2.9	98 ± 2.9
**5%**	**DAPAR**	10 ± 0	0.3 ± 0.5	189.7 ± 0.5	0 ± 0	100 ± 0	99.9 ± 0.3	97.7 ± 4.5	98.8 ± 2.4	98.7 ± 2.5
	**MI4P**	10 ± 0	0.3 ± 0.5	189.7 ± 0.5	0 ± 0	100 ± 0	99.9 ± 0.3	97.7 ± 4.5	98.8 ± 2.4	98.7 ± 2.5
**10%**	**DAPAR**	10 ± 0	0.3 ± 0.6	189.7 ± 0.6	0 ± 0	100 ± 0	99.8 ± 0.3	97.2 ± 4.9	98.5 ± 2.6	98.5 ± 2.7
	**MI4P**	10 ± 0	0.3 ± 0.6	189.7 ± 0.6	0 ± 0	100 ± 0	99.8 ± 0.3	97.2 ± 4.9	98.5 ± 2.6	98.5 ± 2.7
**15%**	**DAPAR**	10 ± 0.1	0.2 ± 0.6	189.8 ± 0.6	0 ± 0.1	99.9 ± 1	99.9 ± 0.3	97.9 ± 4.7	98.8 ± 2.6	98.8 ± 2.6
	**MI4P**	10 ± 0.1	0.2 ± 0.6	189.8 ± 0.6	0 ± 0.1	99.9 ± 1	99.9 ± 0.3	97.9 ± 4.7	98.8 ± 2.6	98.8 ± 2.6
**20%**	**DAPAR**	9.9 ± 0.2	0.4 ± 0.7	189.6 ± 0.7	0.1 ± 0.2	99.4 ± 2.4	99.8 ± 0.4	96.2 ± 5.8	97.6 ± 3.3	97.6 ± 3.3
	**MI4P**	9.9 ± 0.2	0.4 ± 0.7	189.6 ± 0.7	0.1 ± 0.2	99.4 ± 2.4	99.8 ± 0.4	96.2 ± 5.8	97.6 ± 3.3	97.6 ± 3.3
**25%**	**DAPAR**	9.8 ± 0.5	0.9 ± 1	189.1 ± 1	0.2 ± 0.5	97.7 ± 4.7	99.5 ± 0.5	92.7 ± 7.8	94.9 ± 4.7	94.8 ± 4.8
	**MI4P**	9.8 ± 0.5	0.9 ± 1	189.1 ± 1	0.2 ± 0.5	97.7 ± 4.7	99.5 ± 0.5	92.7 ± 7.8	94.9 ± 4.7	94.8 ± 4.8

S3 Table. Performance evaluation on the first set of MAR simulations imputed using k-nearest neighbours. Results are provided as mean ± standard deviation over the 100 simulated datasets for each indicator of performance.
