# Supplementary material for: Accounting for multiple imputation-induced variability for differential analysis in mass spectrometry-based label-free quantitative proteomics
Source: PLoS Comput Biol. 2022 Aug 29;18(8):e1010420. doi: 10.1371/journal.pcbi.1010420 (PMC9462777; doi:10.1371/journal.pcbi.1010420)
Supplement: S4 Table — Results are provided as mean ± standard deviation over the 100 simulated datasets for each indicator of performance. (PDF) [file pcbi.1010420.s004.pdf]

%MV	Method	True positives	False positives	True negatives	False negatives	Sensitivity (%)	Specificity (%)	Precision (%)	F-score (%)	MCC (%)
**1%**	**DAPAR**	10 ± 0.2	0.2 ± 0.4	189.8 ± 0.4	0 ± 0.2	99.8 ± 2	99.9 ± 0.2	98.4 ± 3.7	99 ± 2.2	99 ± 2.2
	**MI4P**	9.9 ± 0.3	0.2 ± 0.4	189.8 ± 0.4	0.1 ± 0.3	99.3 ± 2.9	99.9 ± 0.2	98.3 ± 4	98.7 ± 2.8	98.7 ± 2.8
**5%**	**DAPAR**	10 ± 0.2	0.2 ± 0.4	189.8 ± 0.4	0 ± 0.2	99.6 ± 2	99.9 ± 0.2	98.6 ± 3.7	99 ± 2.1	99 ± 2.2
	**MI4P**	9.7 ± 0.5	0.2 ± 0.4	189.8 ± 0.4	0.3 ± 0.5	96.9 ± 5.4	99.9 ± 0.2	97.9 ± 4.1	97.3 ± 3.4	97.2 ± 3.5
**10%**	**DAPAR**	10 ± 0	0.2 ± 0.5	189.8 ± 0.5	0 ± 0	100 ± 0	99.9 ± 0.2	97.8 ± 4.1	98.9 ± 2.1	98.8 ± 2.2
	**MI4P**	9.6 ± 0.7	0.1 ± 0.3	189.9 ± 0.3	0.4 ± 0.7	95.5 ± 6.9	100 ± 0.1	99.2 ± 2.6	97.2 ± 4	97.1 ± 4
**15%**	**DAPAR**	10 ± 0	0.3 ± 0.6	189.7 ± 0.6	0 ± 0	100 ± 0	99.8 ± 0.3	97.2 ± 4.9	98.5 ± 2.6	98.5 ± 2.7
	**MI4P**	9.2 ± 0.9	0 ± 0.2	190 ± 0.2	0.8 ± 0.9	91.7 ± 8.8	100 ± 0.1	99.6 ± 1.8	95.3 ± 4.9	95.3 ± 4.8
**20%**	**DAPAR**	10 ± 0	0.6 ± 0.8	189.4 ± 0.8	0 ± 0	100 ± 0	99.7 ± 0.4	94.6 ± 6.4	97.1 ± 3.5	97.1 ± 3.6
	**MI4P**	8.9 ± 1	0 ± 0.1	190 ± 0.1	1.1 ± 1	89.1 ± 10.3	100 ± 0.1	99.9 ± 1	93.9 ± 6.1	93.9 ± 5.9
**25%**	**DAPAR**	10 ± 0.1	1.2 ± 1.1	188.8 ± 1.1	0 ± 0.1	99.9 ± 1	99.4 ± 0.6	90.3 ± 8	94.7 ± 4.6	94.6 ± 4.6
	**MI4P**	8.9 ± 1.1	0 ± 0	190 ± 0	1.1 ± 1.1	89.3 ± 11.1	100 ± 0	100 ± 0	94 ± 6.7	94.1 ± 6.4

S4 Table. Performance evaluation on the first set of MAR simulations imputed using Bayesian linear regression. Results are provided as mean ± standard deviation over the 100 simulated datasets for each indicator of performance.
