# Supplementary material for: Accounting for multiple imputation-induced variability for differential analysis in mass spectrometry-based label-free quantitative proteomics
Source: PLoS Comput Biol. 2022 Aug 29;18(8):e1010420. doi: 10.1371/journal.pcbi.1010420 (PMC9462777; doi:10.1371/journal.pcbi.1010420)
Supplement: S5 Table — Results are provided as mean ± standard deviation over the 100 simulated datasets for each indicator of performance. (PDF) [file pcbi.1010420.s005.pdf]

%MV	Method	True positives	False positives	True negatives	False negatives	Sensitivity (%)	Specificity (%)	Precision (%)	F-score (%)	MCC (%)
**1%**	**DAPAR**	10 ± 0	0.5 ± 0.7	189.5 ± 0.7	0 ± 0	100 ± 0	99.7 ± 0.4	95.8 ± 6	97.8 ± 3.3	97.7 ± 3.3
	**MI4P**	10 ± 0	0.5 ± 0.7	189.5 ± 0.7	0 ± 0	100 ± 0	99.7 ± 0.4	95.8 ± 6	97.8 ± 3.3	97.7 ± 3.3
**5%**	**DAPAR**	10 ± 0	0.6 ± 0.8	189.4 ± 0.8	0 ± 0	100 ± 0	99.7 ± 0.4	94.6 ± 6.8	97.1 ± 3.7	97 ± 3.7
	**MI4P**	10 ± 0	0.6 ± 0.8	189.4 ± 0.8	0 ± 0	100 ± 0	99.7 ± 0.4	94.6 ± 6.8	97.1 ± 3.7	97 ± 3.7
**10%**	**DAPAR**	10 ± 0	1 ± 1.1	189 ± 1.1	0 ± 0	100 ± 0	99.5 ± 0.6	91.8 ± 8.3	95.5 ± 4.7	95.5 ± 4.7
	**MI4P**	10 ± 0	1 ± 1.1	189 ± 1.1	0 ± 0	100 ± 0	99.5 ± 0.6	91.8 ± 8.3	95.5 ± 4.7	95.5 ± 4.7
**15%**	**DAPAR**	10 ± 0	1.2 ± 1.2	188.8 ± 1.2	0 ± 0	100 ± 0	99.4 ± 0.6	90.1 ± 8.9	94.5 ± 5.1	94.5 ± 5.1
	**MI4P**	10 ± 0	1.2 ± 1.2	188.8 ± 1.2	0 ± 0	100 ± 0	99.4 ± 0.6	90.1 ± 8.9	94.5 ± 5.1	94.5 ± 5.1
**20%**	**DAPAR**	10 ± 0	1.9 ± 1.5	188 ± 1.5	0 ± 0	100 ± 0	99 ± 0.8	85.1 ± 9.8	91.6 ± 5.9	91.6 ± 5.7
	**MI4P**	10 ± 0	1.9 ± 1.5	188 ± 1.5	0 ± 0	100 ± 0	99 ± 0.8	85.4 ± 9.8	91.8 ± 5.9	91.8 ± 5.7
**25%**	**DAPAR**	10 ± 0.2	2.5 ± 1.6	187.2 ± 1.7	0 ± 0	100 ± 0	98.7 ± 0.9	81 ± 10.5	89.1 ± 6.4	89.2 ± 6.1
	**MI4P**	10 ± 0.2	2.6 ± 1.6	186.8 ± 2	0 ± 0	100 ± 0	98.6 ± 0.9	80.5 ± 10.5	88.8 ± 6.4	88.9 ± 6.2

S5 Table. Performance evaluation on the first set of MAR simulations imputed using principal component analysis. Results are provided as mean ± standard deviation over the 100 simulated datasets for each indicator of performance.
