# Supplementary material for: Accounting for multiple imputation-induced variability for differential analysis in mass spectrometry-based label-free quantitative proteomics
Source: PLoS Comput Biol. 2022 Aug 29;18(8):e1010420. doi: 10.1371/journal.pcbi.1010420 (PMC9462777; doi:10.1371/journal.pcbi.1010420)
Supplement: S6 Table — Results are provided as mean ± standard deviation over the 100 simulated datasets for each indicator of performance. (PDF) [file pcbi.1010420.s006.pdf]

%MV	Method	True positives	False positives	True negatives	False negatives	Sensitivity (%)	Specificity (%)	Precision (%)	F-score (%)	MCC (%)
**1%**	**DAPAR**	10 ± 0	0.5 ± 0.7	189.5 ± 0.7	0 ± 0	100 ± 0	99.8 ± 0.4	96 ± 6	97.9 ± 3.3	97.8 ± 3.3
	**MI4P**	10 ± 0	0.5 ± 0.7	189.5 ± 0.7	0 ± 0	100 ± 0	99.8 ± 0.4	96 ± 6	97.9 ± 3.3	97.8 ± 3.3
**5%**	**DAPAR**	10 ± 0	0.4 ± 0.6	189.6 ± 0.6	0 ± 0	100 ± 0	99.8 ± 0.3	96 ± 5.3	97.9 ± 2.8	97.8 ± 2.9
	**MI4P**	10 ± 0	0.4 ± 0.6	189.6 ± 0.6	0 ± 0	100 ± 0	99.8 ± 0.3	96 ± 5.3	97.9 ± 2.8	97.8 ± 2.9
**10%**	**DAPAR**	10 ± 0	0.5 ± 0.8	189.5 ± 0.8	0 ± 0	100 ± 0	99.7 ± 0.4	95.8 ± 6.7	97.7 ± 3.7	97.7 ± 3.7
	**MI4P**	10 ± 0.1	0.5 ± 0.8	189.5 ± 0.8	0 ± 0.1	99.8 ± 1.4	99.7 ± 0.4	95.9 ± 6.4	97.7 ± 3.6	97.6 ± 3.6
**15%**	**DAPAR**	10 ± 0	0.3 ± 0.6	189.7 ± 0.6	0 ± 0	100 ± 0	99.8 ± 0.3	97.2 ± 5.3	98.5 ± 2.9	98.5 ± 2.9
	**MI4P**	10 ± 0.1	0.4 ± 0.7	189.6 ± 0.7	0 ± 0.1	99.8 ± 1.4	99.8 ± 0.3	96.8 ± 5.5	98.2 ± 3	98.1 ± 3
**20%**	**DAPAR**	10 ± 0.1	0.4 ± 0.6	189.5 ± 0.7	0 ± 0.1	99.8 ± 1.4	99.8 ± 0.3	96.3 ± 5.4	97.9 ± 3	97.9 ± 3.1
	**MI4P**	10 ± 0.1	0.4 ± 0.6	189.4 ± 0.8	0 ± 0.1	99.8 ± 1.4	99.8 ± 0.3	96 ± 5.4	97.8 ± 3	97.7 ± 3.1
**25%**	**DAPAR**	10 ± 0.2	0.3 ± 0.6	189.4 ± 0.9	0 ± 0.1	99.9 ± 1	99.8 ± 0.3	97.5 ± 5	98.6 ± 2.7	98.6 ± 2.8
	**MI4P**	9.9 ± 0.2	0.3 ± 0.6	189.1 ± 1.3	0 ± 0.2	99.7 ± 1.7	99.9 ± 0.3	97.5 ± 4.9	98.5 ± 2.7	98.5 ± 2.8

S6 Table. Performance evaluation on the first set of MAR simulations imputed using random forests. Results are provided as mean ± standard deviation over the 100 simulated datasets for each indicator of performance.
