# Supplementary material for: Accounting for multiple imputation-induced variability for differential analysis in mass spectrometry-based label-free quantitative proteomics
Source: PLoS Comput Biol. 2022 Aug 29;18(8):e1010420. doi: 10.1371/journal.pcbi.1010420 (PMC9462777; doi:10.1371/journal.pcbi.1010420)
Supplement: S7 Table — Results are provided as mean ± standard deviation over the 100 simulated datasets for each indicator of performance. (PDF) [file pcbi.1010420.s007.pdf]

%MV	Method	True positives	False positives	True negatives	False negatives	Sensitivity (%)	Specificity (%)	Precision (%)	F-score (%)	MCC (%)
**1%**	**DAPAR**	80.8 ± 11.4	1.9 ± 1.5	798.1 ± 1.5	119.2 ± 11.4	40.4 ± 5.7	99.8 ± 0.2	97.8 ± 1.6	56.9 ± 5.9	58.2 ± 4.5
	**MI4P**	166.9 ± 5	6.3 ± 2.7	793.7 ± 2.7	33.1 ± 5	83.4 ± 2.5	99.2 ± 0.3	96.4 ± 1.4	89.4 ± 1.5	87.4 ± 1.6
**5%**	**DAPAR**	80.8 ± 12.1	2.4 ± 1.8	797.6 ± 1.8	119.2 ± 12.1	40.4 ± 6.1	99.7 ± 0.2	97.3 ± 1.9	56.8 ± 6.1	58 ± 4.6
	**MI4P**	164.2 ± 6.1	6.1 ± 3.5	793.9 ± 3.5	35.8 ± 6.1	82.1 ± 3	99.2 ± 0.4	96.5 ± 1.9	88.7 ± 1.5	86.6 ± 1.6
**10%**	**DAPAR**	78.8 ± 11.9	2.4 ± 1.6	797.6 ± 1.6	121.2 ± 11.9	39.4 ± 5.9	99.7 ± 0.2	97.1 ± 1.8	55.8 ± 6.1	57.1 ± 4.7
	**MI4P**	160.7 ± 7.8	5.6 ± 3.8	794.4 ± 3.8	39.3 ± 7.8	80.4 ± 3.9	99.3 ± 0.5	96.7 ± 2.1	87.7 ± 1.9	85.6 ± 2
**15%**	**DAPAR**	80.3 ± 11.4	3.3 ± 1.9	796.7 ± 1.9	119.7 ± 11.4	40.1 ± 5.7	99.6 ± 0.2	96.1 ± 2.1	56.4 ± 5.8	57.3 ± 4.6
	**MI4P**	159 ± 8.8	6.7 ± 5.1	793.3 ± 5.1	41 ± 8.8	79.5 ± 4.4	99.2 ± 0.6	96.2 ± 2.7	86.9 ± 2.1	84.7 ± 2.2
**20%**	**DAPAR**	81.3 ± 11.6	4 ± 2.1	796 ± 2.1	118.7 ± 11.6	40.7 ± 5.8	99.5 ± 0.3	95.4 ± 2.4	56.8 ± 5.9	57.4 ± 4.7
	**MI4P**	158 ± 9.8	7.2 ± 5.4	792.8 ± 5.4	42 ± 9.8	79 ± 4.9	99.1 ± 0.7	95.8 ± 2.9	86.5 ± 2.3	84.2 ± 2.3
**25%**	**DAPAR**	82.5 ± 12.3	4.7 ± 2.7	795.3 ± 2.7	117.5 ± 12.3	41.2 ± 6.2	99.4 ± 0.3	94.7 ± 2.8	57.2 ± 6	57.5 ± 4.8
	**MI4P**	154.5 ± 10.4	6.9 ± 6.2	793.1 ± 6.2	45.5 ± 10.4	77.3 ± 5.2	99.1 ± 0.8	96 ± 3.3	85.4 ± 2.5	83.1 ± 2.4

S7 Table. Performance evaluation on the second set of MAR simulations imputed using maximum likelihood estimation. Results are provided as mean ± standard deviation over the 100 simulated datasets for each indicator of performance.
