# Supplementary material for: Accounting for multiple imputation-induced variability for differential analysis in mass spectrometry-based label-free quantitative proteomics
Source: PLoS Comput Biol. 2022 Aug 29;18(8):e1010420. doi: 10.1371/journal.pcbi.1010420 (PMC9462777; doi:10.1371/journal.pcbi.1010420)
Supplement: S8 Table — Results are provided as mean ± standard deviation over the 100 simulated datasets for each indicator of performance. (PDF) [file pcbi.1010420.s008.pdf]

%MV	Method	True positives	False positives	True negatives	False negatives	Sensitivity (%)	Specificity (%)	Precision (%)	F-score (%)	MCC (%)
**1%**	**DAPAR**	80.5 ± 12.1	1.8 ± 1.4	798.2 ± 1.4	119.5 ± 12.1	40.2 ± 6	99.8 ± 0.2	97.9 ± 1.6	56.8 ± 6.3	58.1 ± 4.9
	**MI4P**	167.9 ± 4.8	6.6 ± 2.5	793.4 ± 2.5	32 ± 4.8	84 ± 2.4	99.2 ± 0.3	96.2 ± 1.4	89.7 ± 1.4	87.6 ± 1.7
**5%**	**DAPAR**	79.6 ± 12.4	1.9 ± 1.7	798.1 ± 1.7	120.4 ± 12.4	39.8 ± 6.2	99.8 ± 0.2	97.8 ± 1.9	56.2 ± 6.5	57.7 ± 5
	**MI4P**	169.6 ± 4.3	6.7 ± 2.8	793.3 ± 2.8	30.4 ± 4.3	84.8 ± 2.2	99.2 ± 0.4	96.2 ± 1.5	90.1 ± 1.4	88.1 ± 1.6
**10%**	**DAPAR**	78.2 ± 13.5	2 ± 1.7	798 ± 1.7	121.8 ± 13.5	39.1 ± 6.8	99.8 ± 0.2	97.7 ± 1.8	55.5 ± 7.1	57.1 ± 5.4
	**MI4P**	170.8 ± 4.3	6.3 ± 2.8	793.7 ± 2.8	29.2 ± 4.3	85.4 ± 2.2	99.2 ± 0.4	96.5 ± 1.5	90.6 ± 1.4	88.7 ± 1.6
**15%**	**DAPAR**	79 ± 14.1	2 ± 1.7	798 ± 1.7	121 ± 14.1	39.5 ± 7	99.8 ± 0.2	97.6 ± 1.8	55.9 ± 7.3	57.4 ± 5.6
	**MI4P**	171.6 ± 4.5	6.2 ± 3.1	793.8 ± 3.1	28.4 ± 4.5	85.8 ± 2.2	99.2 ± 0.4	96.5 ± 1.7	90.8 ± 1.4	89 ± 1.7
**20%**	**DAPAR**	77.2 ± 16.8	1.9 ± 1.6	798.1 ± 1.6	122.8 ± 16.8	38.6 ± 8.4	99.8 ± 0.2	97.7 ± 1.9	54.7 ± 9.8	56.4 ± 7.9
	**MI4P**	171.1 ± 4.7	5.7 ± 2.7	794.3 ± 2.7	28.9 ± 4.7	85.5 ± 2.3	99.3 ± 0.3	96.8 ± 1.5	90.8 ± 1.4	89 ± 1.7
**25%**	**DAPAR**	74.4 ± 16.8	1.8 ± 1.7	798.2 ± 1.7	125.6 ± 16.8	37.2 ± 8.4	99.8 ± 0.2	97.7 ± 1.9	53.3 ± 9.8	55.3 ± 7.8
	**MI4P**	170.3 ± 4.9	5.9 ± 2.9	794.1 ± 2.9	29.7 ± 4.9	85.1 ± 2.5	99.3 ± 0.4	96.7 ± 1.6	90.5 ± 1.5	88.6 ± 1.8

S8 Table. Performance evaluation on the second set of MAR simulations imputed using k-nearest neighbours. Results are provided as mean ± standard deviation over the 100 simulated datasets for each indicator of performance.
