# Supplementary material for: Accounting for multiple imputation-induced variability for differential analysis in mass spectrometry-based label-free quantitative proteomics
Source: PLoS Comput Biol. 2022 Aug 29;18(8):e1010420. doi: 10.1371/journal.pcbi.1010420 (PMC9462777; doi:10.1371/journal.pcbi.1010420)
Supplement: S9 Table — Results are provided as mean ± standard deviation over the 100 simulated datasets for each indicator of performance. (PDF) [file pcbi.1010420.s009.pdf]

| %MV | Method | True positives | False positives | True negatives | False negatives | Sensitivity (%) | Specificity (%) | Precision (%) | F-score (%) | MCC (%) |
|---|---|---|---|---|---|---|---|---|---|---|
| **1%** | **DAPAR** | 80.7 ± 11.9 | 1.9 ± 1.6 | 798.1 ± 1.6 | 119.3 ± 11.9 | 40.4 ± 6 | 99.8 ± 0.2 | 97.8 ± 1.8 | 56.8 ± 6.1 | 58.2 ± 4.7 |
| | **MI4P** | 165.7 ± 5 | 5.4 ± 2.4 | 794.6 ± 2.4 | 34.3 ± 5 | 82.8 ± 2.5 | 99.3 ± 0.3 | 96.9 ± 1.3 | 89.3 ± 1.5 | 87.3 ± 1.7 |
| **5%** | **DAPAR** | 80.5 ± 12.5 | 2.3 ± 1.7 | 797.7 ± 1.7 | 119.5 ± 12.5 | 40.3 ± 6.2 | 99.7 ± 0.2 | 97.3 ± 1.8 | 56.6 ± 6.4 | 57.9 ± 4.9 |
| | **MI4P** | 157.3 ± 5.5 | 2.5 ± 1.7 | 797.5 ± 1.7 | 42.6 ± 5.5 | 78.7 ± 2.8 | 99.7 ± 0.2 | 98.5 ± 1 | 87.4 ± 1.7 | 85.5 ± 1.7 |
| **10%** | **DAPAR** | 79.6 ± 12.8 | 2.7 ± 2 | 797.3 ± 2 | 120.4 ± 12.8 | 39.8 ± 6.4 | 99.7 ± 0.2 | 96.9 ± 2.1 | 56.1 ± 6.5 | 57.3 ± 5 |
| | **MI4P** | 156.2 ± 5.7 | 2.4 ± 1.6 | 797.6 ± 1.6 | 43.8 ± 5.7 | 78.1 ± 2.8 | 99.7 ± 0.2 | 98.5 ± 1 | 87.1 ± 1.8 | 85.2 ± 1.9 |
| **15%** | **DAPAR** | 80.6 ± 15 | 3.2 ± 2.4 | 796.8 ± 2.4 | 119.4 ± 15 | 40.3 ± 7.5 | 99.6 ± 0.3 | 96.3 ± 2.5 | 56.3 ± 8.3 | 57.3 ± 6.6 |
| | **MI4P** | 150.7 ± 6.7 | 1.6 ± 1.2 | 798.4 ± 1.2 | 49.3 ± 6.7 | 75.3 ± 3.4 | 99.8 ± 0.1 | 98.9 ± 0.8 | 85.5 ± 2.2 | 83.6 ± 2.2 |
| **20%** | **DAPAR** | 80.5 ± 15.3 | 3.9 ± 2.6 | 796.1 ± 2.6 | 119.5 ± 15.3 | 40.3 ± 7.6 | 99.5 ± 0.3 | 95.5 ± 2.7 | 56.2 ± 8.1 | 57 ± 6.3 |
| | **MI4P** | 144 ± 6.9 | 0.9 ± 1 | 799.1 ± 1 | 56 ± 6.9 | 72 ± 3.4 | 99.9 ± 0.1 | 99.4 ± 0.7 | 83.4 ± 2.3 | 81.7 ± 2.3 |
| **25%** | **DAPAR** | 79.7 ± 17.6 | 4.6 ± 3.2 | 795.4 ± 3.2 | 120.3 ± 17.6 | 39.9 ± 8.8 | 99.4 ± 0.4 | 94.8 ± 2.8 | 55.5 ± 9.5 | 56.3 ± 7.3 |
| | **MI4P** | 137.2 ± 6.7 | 0.6 ± 0.8 | 799.4 ± 0.8 | 62.8 ± 6.7 | 68.6 ± 3.3 | 99.9 ± 0.1 | 99.6 ± 0.6 | 81.2 ± 2.4 | 79.5 ± 2.3 |

**S9 Table. Performance evaluation on the second set of MAR simulations imputed using Bayesian linear regression.** Results are provided as mean ± standard deviation over the 100 simulated datasets for each indicator of performance.
