# Supplementary material for: Accounting for multiple imputation-induced variability for differential analysis in mass spectrometry-based label-free quantitative proteomics
Source: PLoS Comput Biol. 2022 Aug 29;18(8):e1010420. doi: 10.1371/journal.pcbi.1010420 (PMC9462777; doi:10.1371/journal.pcbi.1010420)
Supplement: S10 Table — Results are provided as mean ± standard deviation over the 100 simulated datasets for each indicator of performance. (PDF) [file pcbi.1010420.s010.pdf]

%MV	Method	True positives	False positives	True negatives	False negatives	Sensitivity (%)	Specificity (%)	Precision (%)	F-score (%)	MCC (%)
**1%**	**DAPAR**	80.6 ± 11.8	1.9 ± 1.5	798.1 ± 1.5	119.4 ± 11.8	40.3 ± 5.9	99.8 ± 0.2	97.8 ± 1.7	56.8 ± 6.1	58.1 ± 4.8
	**MI4P**	168.1 ± 4.8	6.8 ± 2.7	793.2 ± 2.7	31.9 ± 4.8	84 ± 2.4	99.2 ± 0.3	96.1 ± 1.5	89.7 ± 1.5	87.6 ± 1.7
**5%**	**DAPAR**	80.9 ± 12.6	2.4 ± 1.8	797.6 ± 1.8	119.1 ± 12.6	40.4 ± 6.3	99.7 ± 0.2	97.2 ± 2	56.8 ± 6.5	58 ± 5
	**MI4P**	170 ± 4.6	7.6 ± 2.9	792.5 ± 2.9	30 ± 4.6	85 ± 2.3	99.1 ± 0.4	95.8 ± 1.6	90 ± 1.4	88 ± 1.6
**10%**	**DAPAR**	79.9 ± 13	2.8 ± 1.9	797.2 ± 1.9	120.1 ± 13	40 ± 6.5	99.7 ± 0.2	96.8 ± 2	56.2 ± 6.6	57.4 ± 5.1
	**MI4P**	172.1 ± 4.6	8.2 ± 3	791.8 ± 3	27.9 ± 4.6	86.1 ± 2.3	99 ± 0.4	95.5 ± 1.5	90.5 ± 1.4	88.5 ± 1.6
**15%**	**DAPAR**	81.8 ± 12.9	3.6 ± 2.5	796.4 ± 2.5	118.2 ± 12.9	40.9 ± 6.4	99.6 ± 0.3	95.9 ± 2.5	57 ± 6.5	57.8 ± 5.1
	**MI4P**	174.2 ± 4	9.4 ± 3.6	790.6 ± 3.6	25.8 ± 4	87.1 ± 2	98.8 ± 0.5	94.9 ± 1.9	90.8 ± 1.3	88.8 ± 1.6
**20%**	**DAPAR**	82.1 ± 15.4	4.4 ± 2.6	795.6 ± 2.6	117.9 ± 15.4	41 ± 7.7	99.5 ± 0.3	95.1 ± 2.7	56.8 ± 8	57.4 ± 6.2
	**MI4P**	175.6 ± 4.1	11.3 ± 4.1	788.7 ± 4.1	24.4 ± 4.1	87.8 ± 2.1	98.6 ± 0.5	94 ± 2	90.8 ± 1.5	88.7 ± 1.8
**25%**	**DAPAR**	83.3 ± 14.6	5.3 ± 2.9	794.7 ± 2.9	116.7 ± 14.6	41.6 ± 7.3	99.3 ± 0.4	94.1 ± 2.8	57.3 ± 7.3	57.5 ± 5.8
	**MI4P**	176.3 ± 4.5	13 ± 3.8	787 ± 3.8	23.7 ± 4.5	88.1 ± 2.3	98.4 ± 0.5	93.2 ± 1.9	90.6 ± 1.5	88.4 ± 1.8

S10 Table. Performance evaluation on the second set of MAR simulations imputed using principal component analysis. Results are provided as mean ± standard deviation over the 100 simulated datasets for each indicator of performance.
