# Supplementary material for: Accounting for multiple imputation-induced variability for differential analysis in mass spectrometry-based label-free quantitative proteomics
Source: PLoS Comput Biol. 2022 Aug 29;18(8):e1010420. doi: 10.1371/journal.pcbi.1010420 (PMC9462777; doi:10.1371/journal.pcbi.1010420)
Supplement: S11 Table — Results are provided as mean ± standard deviation over the 100 simulated datasets for each indicator of performance. (PDF) [file pcbi.1010420.s011.pdf]

%MV	Method	True positives	False positives	True negatives	False negatives	Sensitivity (%)	Specificity (%)	Precision (%)	F-score (%)	MCC (%)
**1%**	**DAPAR**	80.8 ± 11.7	1.9 ± 1.5	798.1 ± 1.5	119.2 ± 11.7	40.4 ± 5.8	99.8 ± 0.2	97.8 ± 1.7	56.9 ± 6	58.2 ± 4.7
	**MI4P**	168 ± 4.7	6.8 ± 2.7	793.2 ± 2.7	32 ± 4.7	84 ± 2.4	99.2 ± 0.3	96.1 ± 1.4	89.6 ± 1.4	87.6 ± 1.7
**5%**	**DAPAR**	80.7 ± 12.7	2.4 ± 1.9	797.6 ± 1.9	119.3 ± 12.7	40.3 ± 6.3	99.7 ± 0.2	97.2 ± 2	56.7 ± 6.5	57.9 ± 5
	**MI4P**	169.9 ± 4.4	7.5 ± 3	792.5 ± 3	30.1 ± 4.4	85 ± 2.2	99.1 ± 0.4	95.8 ± 1.6	90 ± 1.4	88 ± 1.6
**10%**	**DAPAR**	79.9 ± 12.5	2.7 ± 1.8	797.3 ± 1.8	120.1 ± 12.5	40 ± 6.3	99.7 ± 0.2	96.8 ± 2	56.3 ± 6.4	57.5 ± 5
	**MI4P**	171.6 ± 4.6	8.1 ± 3.1	792 ± 3.1	28.4 ± 4.6	85.8 ± 2.3	99 ± 0.4	95.5 ± 1.6	90.4 ± 1.5	88.4 ± 1.7
**15%**	**DAPAR**	81.4 ± 13.8	3.5 ± 2.4	796.5 ± 2.4	118.6 ± 13.8	40.7 ± 6.9	99.6 ± 0.3	96 ± 2.4	56.8 ± 7.1	57.6 ± 5.5
	**MI4P**	173.5 ± 4	9.3 ± 3.8	790.7 ± 3.8	26.5 ± 4	86.8 ± 2	98.8 ± 0.5	94.9 ± 1.9	90.6 ± 1.4	88.6 ± 1.7
**20%**	**DAPAR**	82.1 ± 13.5	4.4 ± 2.6	795.6 ± 2.6	117.9 ± 13.5	41.1 ± 6.8	99.4 ± 0.3	95 ± 2.6	57 ± 6.9	57.5 ± 5.4
	**MI4P**	174.4 ± 4.1	10.9 ± 3.9	789.1 ± 3.9	25.6 ± 4.1	87.2 ± 2	98.6 ± 0.5	94.1 ± 2	90.5 ± 1.4	88.4 ± 1.7
**25%**	**DAPAR**	82.2 ± 16	5 ± 2.9	795 ± 2.9	117.8 ± 16	41.1 ± 8	99.4 ± 0.4	94.4 ± 2.8	56.8 ± 8.5	57.2 ± 6.7
	**MI4P**	174.7 ± 4.5	12.4 ± 4	787.6 ± 4	25.3 ± 4.5	87.3 ± 2.2	98.5 ± 0.5	93.4 ± 1.9	90.3 ± 1.5	88 ± 1.8

S11 Table. Performance evaluation on the second set of MAR simulations imputed using random forests.
Results are provided as mean ± standard deviation over the 100 simulated datasets for each indicator of performance.
