# Supplementary material for: Accounting for multiple imputation-induced variability for differential analysis in mass spectrometry-based label-free quantitative proteomics
Source: PLoS Comput Biol. 2022 Aug 29;18(8):e1010420. doi: 10.1371/journal.pcbi.1010420 (PMC9462777; doi:10.1371/journal.pcbi.1010420)
Supplement: S12 Table — Results are provided as mean ± standard deviation over the 100 simulated datasets for each indicator of performance. (PDF) [file pcbi.1010420.s012.pdf]

%MV	Method	True positives	False positives	True negatives	False negatives	Sensitivity (%)	Specificity (%)	Precision (%)	F-score (%)	MCC (%)
**1%**	**DAPAR**	25.6 ± 10.7	0.5 ± 0.8	799.5 ± 0.8	174.4 ± 10.7	12.8 ± 5.4	99.9 ± 0.1	98.3 ± 2.4	22.2 ± 8.4	31.2 ± 7.4
	**MI4P**	91 ± 10.6	2.7 ± 1.8	797.3 ± 1.8	109 ± 10.6	45.5 ± 5.3	99.7 ± 0.2	97.2 ± 1.8	61.8 ± 4.9	61.9 ± 4
**5%**	**DAPAR**	25.6 ± 10.2	0.4 ± 0.7	799.6 ± 0.7	174.4 ± 10.2	12.8 ± 5.1	99.9 ± 0.1	98.5 ± 2.4	22.3 ± 7.9	31.4 ± 6.8
	**MI4P**	83 ± 13.6	2.1 ± 1.8	797.9 ± 1.8	117 ± 13.6	41.5 ± 6.8	99.7 ± 0.2	97.6 ± 1.9	57.9 ± 6.7	59 ± 5.1
**10%**	**DAPAR**	25.9 ± 10.8	0.6 ± 0.7	799.4 ± 0.7	174.1 ± 10.8	13 ± 5.4	99.9 ± 0.1	96.1 ± 14	22.5 ± 8.6	31.1 ± 8.3
	**MI4P**	80.2 ± 18.2	2.3 ± 2.1	797.7 ± 2.1	119.8 ± 18.2	40.1 ± 9.1	99.7 ± 0.3	97.5 ± 2	56.2 ± 9.2	57.6 ± 6.9
**15%**	**DAPAR**	26.6 ± 11.5	0.8 ± 1	799.2 ± 1	173.4 ± 11.5	13.3 ± 5.7	99.9 ± 0.1	96.5 ± 10.3	23 ± 9	31.5 ± 8.2
	**MI4P**	71.9 ± 22.7	2.1 ± 2.3	797.9 ± 2.3	128.1 ± 22.7	35.9 ± 11.3	99.7 ± 0.3	97.7 ± 2.3	51.4 ± 12.3	54 ± 9.1
**20%**	**DAPAR**	28.5 ± 12.1	1.1 ± 1.3	798.9 ± 1.3	171.5 ± 12.1	14.2 ± 6.1	99.9 ± 0.2	95.4 ± 10.4	24.3 ± 9.3	32.3 ± 8.5
	**MI4P**	67.1 ± 22.4	1.9 ± 2.3	798.1 ± 2.3	132.9 ± 22.4	33.6 ± 11.2	99.8 ± 0.3	97.8 ± 2.3	48.8 ± 12.4	52 ± 9.2
**25%**	**DAPAR**	26.9 ± 12.4	1.3 ± 1.4	798.7 ± 1.4	173.1 ± 12.4	13.4 ± 6.2	99.8 ± 0.2	96.2 ± 4	23 ± 9.7	31.1 ± 8.6
	**MI4P**	61.2 ± 24	2 ± 2.8	798 ± 2.8	138.8 ± 24	30.6 ± 12	99.7 ± 0.4	97.7 ± 2.8	45.2 ± 13.6	49.2 ± 10

S12 Table. Performance evaluation on the third set of MAR simulations imputed using maximum likelihood estimation. Results are provided as mean ± standard deviation over the 100 simulated datasets for each indicator of performance.
