# Supplementary material for: Accounting for multiple imputation-induced variability for differential analysis in mass spectrometry-based label-free quantitative proteomics
Source: PLoS Comput Biol. 2022 Aug 29;18(8):e1010420. doi: 10.1371/journal.pcbi.1010420 (PMC9462777; doi:10.1371/journal.pcbi.1010420)
Supplement: S13 Table — Results are provided as mean ± standard deviation over the 100 simulated datasets for each indicator of performance. (PDF) [file pcbi.1010420.s013.pdf]

%MV	Method	True positives	False positives	True negatives	False negatives	Sensitivity (%)	Specificity (%)	Precision (%)	F-score (%)	MCC (%)
**1%**	**DAPAR**	26 ± 10.4	0.5 ± 0.8	799.5 ± 0.8	174 ± 10.4	13 ± 5.2	99.9 ± 0.1	98.5 ± 2.3	22.5 ± 8.1	31.5 ± 7
	**MI4P**	95.8 ± 9.8	3.1 ± 1.9	796.9 ± 1.9	104.2 ± 9.8	47.9 ± 4.9	99.6 ± 0.2	96.9 ± 1.8	64 ± 4.4	63.6 ± 3.7
**5%**	**DAPAR**	25.4 ± 11.1	0.4 ± 0.7	799.6 ± 0.7	174.6 ± 11.1	12.7 ± 5.5	99.9 ± 0.1	98.5 ± 2.5	22.1 ± 8.7	31.1 ± 7.5
	**MI4P**	98 ± 9.9	2.9 ± 1.8	797.1 ± 1.8	102 ± 9.9	49 ± 4.9	99.6 ± 0.2	97.1 ± 1.7	65 ± 4.4	64.6 ± 3.7
**10%**	**DAPAR**	24.5 ± 10.6	0.6 ± 0.9	799.4 ± 0.9	175.5 ± 10.6	12.3 ± 5.3	99.9 ± 0.1	95.8 ± 14.1	21.4 ± 8.4	30.2 ± 7.9
	**MI4P**	101.1 ± 9.5	3.2 ± 1.8	796.8 ± 1.8	98.9 ± 9.5	50.6 ± 4.8	99.6 ± 0.2	97 ± 1.6	66.3 ± 4.1	65.6 ± 3.5
**15%**	**DAPAR**	25.1 ± 12.2	0.4 ± 0.7	799.6 ± 0.7	174.9 ± 12.2	12.5 ± 6.1	99.9 ± 0.1	96.4 ± 14.1	21.7 ± 9.7	30.4 ± 9.2
	**MI4P**	103.8 ± 10.9	2.6 ± 1.4	797.4 ± 1.4	96.2 ± 10.9	51.9 ± 5.4	99.7 ± 0.2	97.6 ± 1.3	67.6 ± 4.7	66.8 ± 4
**20%**	**DAPAR**	24.7 ± 13.2	0.4 ± 0.7	799.6 ± 0.7	175.3 ± 13.2	12.3 ± 6.6	99.9 ± 0.1	95.6 ± 17.1	21.3 ± 10.4	29.9 ± 10.1
	**MI4P**	106.2 ± 11.9	2.7 ± 1.7	797.3 ± 1.7	93.8 ± 11.9	53.1 ± 5.9	99.7 ± 0.2	97.6 ± 1.4	68.6 ± 5	67.7 ± 4.3
**25%**	**DAPAR**	24.7 ± 12.3	0.6 ± 0.9	799.4 ± 0.9	175.3 ± 12.3	12.3 ± 6.2	99.9 ± 0.1	96.8 ± 10.3	21.4 ± 9.7	30.1 ± 8.9
	**MI4P**	105.4 ± 11.1	2.9 ± 1.9	797.1 ± 1.9	94.6 ± 11.1	52.7 ± 5.5	99.6 ± 0.2	97.4 ± 1.6	68.2 ± 4.7	67.3 ± 4

S13 Table. Performance evaluation on the third set of MAR simulations imputed using k-nearest neighbours. Results are provided as mean ± standard deviation over the 100 simulated datasets for each indicator of performance.
