# Supplementary material for: Accounting for multiple imputation-induced variability for differential analysis in mass spectrometry-based label-free quantitative proteomics
Source: PLoS Comput Biol. 2022 Aug 29;18(8):e1010420. doi: 10.1371/journal.pcbi.1010420 (PMC9462777; doi:10.1371/journal.pcbi.1010420)
Supplement: S14 Table — Results are provided as mean ± standard deviation over the 100 simulated datasets for each indicator of performance. (PDF) [file pcbi.1010420.s014.pdf]

%MV	Method	True positives	False positives	True negatives	False negatives	Sensitivity (%)	Specificity (%)	Precision (%)	F-score (%)	MCC (%)
**1%**	**DAPAR**	25.8 ± 10.6	0.5 ± 0.8	799.5 ± 0.8	174.2 ± 10.6	12.9 ± 5.3	99.9 ± 0.1	98.3 ± 2.5	22.4 ± 8.4	31.3 ± 7.4
	**MI4P**	87.9 ± 9.5	2.2 ± 1.6	797.8 ± 1.6	112.1 ± 9.5	43.9 ± 4.8	99.7 ± 0.2	97.6 ± 1.7	60.4 ± 4.5	60.9 ± 3.7
**5%**	**DAPAR**	25.6 ± 10.7	0.5 ± 0.7	799.5 ± 0.7	174.4 ± 10.7	12.8 ± 5.4	99.9 ± 0.1	98.4 ± 2.4	22.3 ± 8.4	31.3 ± 7.3
	**MI4P**	63.1 ± 10.4	0.5 ± 0.7	799.5 ± 0.7	136.9 ± 10.4	31.5 ± 5.2	99.9 ± 0.1	99.2 ± 1.1	47.6 ± 6.1	51.4 ± 4.6
**10%**	**DAPAR**	24.4 ± 11.5	0.6 ± 0.8	799.4 ± 0.8	175.6 ± 11.5	12.2 ± 5.7	99.9 ± 0.1	96 ± 14.1	21.2 ± 9.2	29.9 ± 8.8
	**MI4P**	37.2 ± 11.3	0.1 ± 0.3	799.9 ± 0.3	162.8 ± 11.3	18.6 ± 5.6	100 ± 0	99.7 ± 0.9	31 ± 8.1	38.8 ± 6.4
**15%**	**DAPAR**	24.9 ± 12.4	0.7 ± 0.9	799.3 ± 0.9	175.1 ± 12.4	12.5 ± 6.2	99.9 ± 0.1	95.7 ± 14	21.6 ± 9.7	30.1 ± 9.2
	**MI4P**	17.6 ± 11.7	0 ± 0.2	800 ± 0.2	182.4 ± 11.7	8.8 ± 5.8	100 ± 0	92.9 ± 25.6	15.6 ± 9.8	24.5 ± 11.1
**20%**	**DAPAR**	23.3 ± 12.4	0.7 ± 1	799.3 ± 1	176.7 ± 12.4	11.6 ± 6.2	99.9 ± 0.1	96.3 ± 10.5	20.2 ± 9.8	28.9 ± 9.2
	**MI4P**	6.4 ± 6.9	0 ± 0	800 ± 0	193.6 ± 6.9	3.2 ± 3.5	100 ± 0	74 ± 44.1	6 ± 6.3	12.8 ± 9.8
**25%**	**DAPAR**	24.1 ± 11.8	0.8 ± 1.2	799.2 ± 1.2	175.8 ± 11.8	12.1 ± 5.9	99.9 ± 0.1	97.4 ± 3.5	21 ± 9.3	29.7 ± 8.2
	**MI4P**	1.7 ± 3.2	0 ± 0	800 ± 0	198.3 ± 3.2	0.9 ± 1.6	100 ± 0	43 ± 49.8	1.7 ± 3	5 ± 6.8

S14 Table. Performance evaluation on the third set of MAR simulations imputed using Bayesian linear regression. Results are provided as mean ± standard deviation over the 100 simulated datasets for each indicator of performance.
