# Supplementary material for: Accounting for multiple imputation-induced variability for differential analysis in mass spectrometry-based label-free quantitative proteomics
Source: PLoS Comput Biol. 2022 Aug 29;18(8):e1010420. doi: 10.1371/journal.pcbi.1010420 (PMC9462777; doi:10.1371/journal.pcbi.1010420)
Supplement: S15 Table — Results are provided as mean ± standard deviation over the 100 simulated datasets for each indicator of performance. (PDF) [file pcbi.1010420.s015.pdf]

%MV	Method	True positives	False positives	True negatives	False negatives	Sensitivity (%)	Specificity (%)	Precision (%)	F-score (%)	MCC (%)
**1%**	**DAPAR**	25.8 ± 10.2	0.5 ± 0.8	799.5 ± 0.8	174.2 ± 10.2	12.9 ± 5.1	99.9 ± 0.1	98.3 ± 2.4	22.4 ± 8	31.4 ± 7
	**MI4P**	95.7 ± 9.9	3.2 ± 1.8	796.8 ± 1.8	104.3 ± 9.9	47.9 ± 4.9	99.6 ± 0.2	96.8 ± 1.7	63.9 ± 4.4	63.5 ± 3.7
**5%**	**DAPAR**	24.9 ± 10.4	0.5 ± 0.7	799.5 ± 0.7	175.2 ± 10.4	12.4 ± 5.2	99.9 ± 0.1	98.2 ± 2.5	21.7 ± 8.3	30.6 ± 7.5
	**MI4P**	97.7 ± 9.5	3 ± 1.8	797 ± 1.8	102.3 ± 9.5	48.8 ± 4.7	99.6 ± 0.2	97 ± 1.7	64.8 ± 4.2	64.4 ± 3.6
**10%**	**DAPAR**	24.5 ± 10.6	0.6 ± 0.9	799.4 ± 0.9	175.5 ± 10.6	12.3 ± 5.3	99.9 ± 0.1	95.8 ± 14.1	21.4 ± 8.4	30.2 ± 7.9
	**MI4P**	101.1 ± 9.5	3.2 ± 1.8	796.8 ± 1.8	98.9 ± 9.5	50.6 ± 4.8	99.6 ± 0.2	97 ± 1.6	66.3 ± 4.1	65.6 ± 3.5
**15%**	**DAPAR**	24.2 ± 12.4	0.7 ± 0.9	799.3 ± 0.9	175.8 ± 12.4	12.1 ± 6.2	99.9 ± 0.1	95.7 ± 14	21 ± 9.7	29.6 ± 9.1
	**MI4P**	104.6 ± 10.1	3.4 ± 2.1	796.6 ± 2.1	95.4 ± 10.1	52.3 ± 5.1	99.6 ± 0.3	96.9 ± 1.8	67.8 ± 4.3	66.8 ± 3.7
**20%**	**DAPAR**	23.6 ± 12.2	0.7 ± 0.9	799.3 ± 0.9	176.4 ± 12.2	11.8 ± 6.1	99.9 ± 0.1	94.7 ± 17.1	20.5 ± 9.7	29 ± 9.7
	**MI4P**	110 ± 10.1	3.7 ± 2.1	796.3 ± 2.1	90 ± 10.1	55 ± 5.1	99.5 ± 0.3	96.8 ± 1.7	70 ± 4.2	68.7 ± 3.6
**25%**	**DAPAR**	24.7 ± 11.3	0.8 ± 1.2	799.2 ± 1.2	175.3 ± 11.3	12.3 ± 5.7	99.9 ± 0.1	97.2 ± 3.6	21.4 ± 8.9	30.2 ± 7.7
	**MI4P**	113.6 ± 9.3	4.4 ± 2.3	795.6 ± 2.3	86.4 ± 9.3	56.8 ± 4.6	99.4 ± 0.3	96.3 ± 1.7	71.3 ± 3.6	69.7 ± 3.2

S15 Table. Performance evaluation on the third set of MAR simulations imputed using principal component analysis. Results are provided as mean ± standard deviation over the 100 simulated datasets for each indicator of performance.
