# Supplementary material for: Accounting for multiple imputation-induced variability for differential analysis in mass spectrometry-based label-free quantitative proteomics
Source: PLoS Comput Biol. 2022 Aug 29;18(8):e1010420. doi: 10.1371/journal.pcbi.1010420 (PMC9462777; doi:10.1371/journal.pcbi.1010420)
Supplement: S16 Table — Results are provided as mean ± standard deviation over the 100 simulated datasets for each indicator of performance. (PDF) [file pcbi.1010420.s016.pdf]

%MV	Method	True positives	False positives	True negatives	False negatives	Sensitivity (%)	Specificity (%)	Precision (%)	F-score (%)	MCC (%)
**1%**	**DAPAR**	25.7 ± 10.2	0.5 ± 0.7	799.5 ± 0.7	174.3 ± 10.2	12.8 ± 5.1	99.9 ± 0.1	98.5 ± 2.3	22.3 ± 8	31.3 ± 7
	**MI4P**	95.8 ± 9.8	3.1 ± 1.9	796.9 ± 1.9	104.2 ± 9.8	47.9 ± 4.9	99.6 ± 0.2	96.9 ± 1.8	63.9 ± 4.4	63.6 ± 3.7
**5%**	**DAPAR**	25.2 ± 10.5	0.5 ± 0.7	799.5 ± 0.7	174.8 ± 10.5	12.6 ± 5.2	99.9 ± 0.1	98.4 ± 2.5	21.9 ± 8.2	31 ± 7.1
	**MI4P**	97.7 ± 9.8	3 ± 1.8	797 ± 1.8	102.3 ± 9.8	48.8 ± 4.9	99.6 ± 0.2	97.1 ± 1.7	64.8 ± 4.3	64.4 ± 3.6
**10%**	**DAPAR**	24.4 ± 11.4	0.5 ± 0.8	799.5 ± 0.8	175.6 ± 11.4	12.2 ± 5.7	99.9 ± 0.1	95.2 ± 17.1	21.2 ± 9.1	29.9 ± 9.1
	**MI4P**	102.2 ± 9.9	2.9 ± 1.7	797.1 ± 1.7	97.8 ± 9.9	51.1 ± 4.9	99.6 ± 0.2	97.3 ± 1.6	66.9 ± 4.3	66.1 ± 3.7
**15%**	**DAPAR**	25.4 ± 12.7	0.5 ± 0.8	799.5 ± 0.8	174.6 ± 12.7	12.7 ± 6.3	99.9 ± 0.1	96.4 ± 14.1	21.9 ± 10	30.5 ± 9.5
	**MI4P**	105.7 ± 10.1	2.7 ± 1.6	797.3 ± 1.6	94.3 ± 10.1	52.8 ± 5.1	99.7 ± 0.2	97.5 ± 1.4	68.4 ± 4.3	67.5 ± 3.7
**20%**	**DAPAR**	25.1 ± 12.5	0.4 ± 0.7	799.5 ± 0.7	174.9 ± 12.5	12.5 ± 6.3	99.9 ± 0.1	95.6 ± 17.1	21.7 ± 9.8	30.4 ± 9.5
	**MI4P**	110.8 ± 10.2	3 ± 1.9	797 ± 1.9	89.2 ± 10.2	55.4 ± 5.1	99.6 ± 0.2	97.4 ± 1.5	70.5 ± 4.1	69.3 ± 3.5
**25%**	**DAPAR**	26.7 ± 12.1	0.7 ± 1	799.3 ± 1	173.3 ± 12.1	13.3 ± 6	99.9 ± 0.1	97.8 ± 3.2	23 ± 9.5	31.6 ± 8.4
	**MI4P**	113.9 ± 9.8	3.4 ± 2	796.6 ± 2	86.1 ± 9.8	57 ± 4.9	99.6 ± 0.3	97.1 ± 1.6	71.7 ± 3.9	70.2 ± 3.4

S16 Table. Performance evaluation on the third set of MAR simulations imputed using random forests. Results are provided as mean ± standard deviation over the 100 simulated datasets for each indicator of performance.
