# Supplementary material for: Accounting for multiple imputation-induced variability for differential analysis in mass spectrometry-based label-free quantitative proteomics
Source: PLoS Comput Biol. 2022 Aug 29;18(8):e1010420. doi: 10.1371/journal.pcbi.1010420 (PMC9462777; doi:10.1371/journal.pcbi.1010420)
Supplement: S17 Table — Results are provided as mean ± standard deviation over the 100 simulated datasets for each indicator of performance. (PDF) [file pcbi.1010420.s017.pdf]

%MV	Method	True positives	False positives	True negatives	False negatives	Sensitivity (%)	Specificity (%)	Precision (%)	F-score (%)	MCC (%)
**1%**	**DAPAR**	123.7 ± 7.7	490.5 ± 17.4	309.5 ± 17.4	76.3 ± 7.7	61.8 ± 3.8	38.7 ± 2.2	20.1 ± 1.1	30.4 ± 1.6	0.4 ± 3.4
	**MI4P**	154.8 ± 6.5	617.2 ± 12	182.8 ± 12	45.2 ± 6.5	77.4 ± 3.3	22.8 ± 1.5	20 ± 0.7	31.8 ± 1.2	0.2 ± 3.4
**5%**	**DAPAR**	125.1 ± 6.6	495.7 ± 17.1	303.9 ± 17	74.9 ± 6.6	62.5 ± 3.3	38 ± 2.1	20.1 ± 0.9	30.5 ± 1.3	0.5 ± 2.8
	**MI4P**	154.4 ± 5.3	613.1 ± 13.5	186.1 ± 12.8	45.5 ± 5.3	77.2 ± 2.7	23.3 ± 1.6	20.1 ± 0.7	31.9 ± 1	0.5 ± 2.9
**10%**	**DAPAR**	123.9 ± 6.9	477.8 ± 18.3	312.6 ± 17.6	76.1 ± 6.9	61.9 ± 3.5	39.6 ± 2.2	20.6 ± 1.1	30.9 ± 1.6	1.2 ± 3.3
	**MI4P**	152.9 ± 6.1	590.3 ± 17.6	193.2 ± 15.6	47.1 ± 6.1	76.5 ± 3.1	24.7 ± 2	20.6 ± 0.8	32.4 ± 1.2	1 ± 3.3
**15%**	**DAPAR**	124.9 ± 7.2	436 ± 18.6	317.4 ± 18.3	75.1 ± 7.2	62.5 ± 3.6	42.1 ± 2.3	22.3 ± 1.1	32.8 ± 1.7	3.8 ± 3.2
	**MI4P**	153.3 ± 6	540.4 ± 18.8	201.1 ± 14.8	46.7 ± 6	76.7 ± 3	27.1 ± 1.9	22.1 ± 0.8	34.3 ± 1.2	3.5 ± 3.2
**20%**	**DAPAR**	124.4 ± 7.6	396.1 ± 19.1	326.7 ± 23.9	75.6 ± 7.6	62.2 ± 3.8	45.2 ± 2.8	23.9 ± 1.1	34.5 ± 1.6	6.1 ± 3.1
	**MI4P**	153.3 ± 5.9	492.2 ± 21.7	206.6 ± 18.9	46.7 ± 5.9	76.6 ± 2.9	29.5 ± 2.2	23.8 ± 0.9	36.3 ± 1.2	5.7 ± 2.9
**25%**	**DAPAR**	119.7 ± 7.6	349.3 ± 19.3	324.4 ± 25.3	80.3 ± 7.6	59.8 ± 3.8	48.1 ± 2.9	25.5 ± 1.3	35.8 ± 1.8	6.7 ± 3.6
	**MI4P**	146.9 ± 6.9	439.9 ± 23.9	200 ± 28.1	53.1 ± 6.9	73.4 ± 3.5	31.1 ± 2.8	25.1 ± 1.1	37.3 ± 1.5	4.2 ± 4.4

S17 Table. Performance evaluation on the first set of MCAR + MNAR simulations imputed using maximum likelihood estimation. Results are provided as mean ± standard deviation over the 100 simulated datasets for each indicator of performance.
