# Supplementary material for: Accounting for multiple imputation-induced variability for differential analysis in mass spectrometry-based label-free quantitative proteomics
Source: PLoS Comput Biol. 2022 Aug 29;18(8):e1010420. doi: 10.1371/journal.pcbi.1010420 (PMC9462777; doi:10.1371/journal.pcbi.1010420)
Supplement: S18 Table — Results are provided as mean ± standard deviation over the 100 simulated datasets for each indicator of performance. (PDF) [file pcbi.1010420.s018.pdf]

%MV	Method	True positives	False positives	True negatives	False negatives	Sensitivity (%)	Specificity (%)	Precision (%)	F-score (%)	MCC (%)
**1%**	**DAPAR**	306.5 ± 10	6152.2 ± 65.7	3847.8 ± 65.7	193.5 ± 10	61.3 ± 2	38.5 ± 0.7	4.7 ± 0.1	8.8 ± 0.3	-0.1 ± 0.9
	**MI4P**	384.5 ± 9.5	7711 ± 46.5	2289 ± 46.5	115.5 ± 9.5	76.9 ± 1.9	22.9 ± 0.5	4.7 ± 0.1	8.9 ± 0.2	-0.1 ± 1
**5%**	**DAPAR**	311.7 ± 11	6133.4 ± 64.2	3847 ± 66.3	188.3 ± 11	62.3 ± 2.2	38.5 ± 0.6	4.8 ± 0.2	9 ± 0.3	0.4 ± 1
	**MI4P**	384.6 ± 9.2	7631.6 ± 67.8	2336.6 ± 55.5	115.4 ± 9.2	76.9 ± 1.8	23.4 ± 0.6	4.8 ± 0.1	9 ± 0.2	0.2 ± 0.9
**10%**	**DAPAR**	311.9 ± 10.8	6007.3 ± 91.1	3862.8 ± 91	188.1 ± 10.8	62.4 ± 2.2	39.1 ± 0.9	4.9 ± 0.2	9.1 ± 0.3	0.7 ± 1
	**MI4P**	384 ± 9.7	7400.3 ± 127.1	2397.3 ± 89.2	116 ± 9.7	76.8 ± 1.9	24.5 ± 0.9	4.9 ± 0.1	9.3 ± 0.2	0.6 ± 0.9
**15%**	**DAPAR**	315.6 ± 11.5	5566.6 ± 125.6	3903.5 ± 153.6	184.4 ± 11.5	63.1 ± 2.3	41.2 ± 1.3	5.4 ± 0.2	9.9 ± 0.3	1.9 ± 1
	**MI4P**	384.2 ± 11	6842.1 ± 172.1	2470.2 ± 117.6	115.8 ± 11	76.8 ± 2.2	26.5 ± 1	5.3 ± 0.2	9.9 ± 0.3	1.7 ± 1.1
**20%**	**DAPAR**	315.6 ± 13.2	5157.3 ± 123.1	3916.9 ± 200.8	184.4 ± 13.2	63.1 ± 2.6	43.1 ± 1.6	5.8 ± 0.2	10.6 ± 0.4	2.8 ± 1.1
	**MI4P**	384.4 ± 10.7	6312.7 ± 210.3	2493.2 ± 164.8	115.6 ± 10.7	76.9 ± 2.1	28.3 ± 1.2	5.7 ± 0.2	10.7 ± 0.3	2.6 ± 1.1
**25%**	**DAPAR**	310.1 ± 14.3	4696.8 ± 117.4	3831.4 ± 260.5	189.9 ± 14.3	62 ± 2.9	44.9 ± 1.9	6.2 ± 0.2	11.3 ± 0.5	3.2 ± 1.3
	**MI4P**	370.2 ± 12.6	5752.6 ± 224.1	2449.7 ± 248.5	129.8 ± 12.6	74 ± 2.5	29.8 ± 1.7	6.1 ± 0.2	11.2 ± 0.3	1.9 ± 1.6

S18 Table. Performance evaluation on the second set of MCAR + MNAR simulations imputed using maximum likelihood estimation. Results are provided as mean ± standard deviation over the 100 simulated datasets for each indicator of performance.
