# Supplementary material for: Accounting for multiple imputation-induced variability for differential analysis in mass spectrometry-based label-free quantitative proteomics
Source: PLoS Comput Biol. 2022 Aug 29;18(8):e1010420. doi: 10.1371/journal.pcbi.1010420 (PMC9462777; doi:10.1371/journal.pcbi.1010420)
Supplement: S19 Table — Missing values were imputed using the maximum likelihood estimation method. (PDF) [file pcbi.1010420.s019.pdf]

Condition (vs 10fmol)	Method	True positives	False positives	True negatives	False negatives	Sensitivity (%)	Specificity (%)	Precision (%)	F-score (%)	MCC (%)
**0.05fmol**	**DAPAR**	132	3677	10507	5	96.4	74.1	3.5	6.7	15.5
	**MI4P**	129	2095	12089	8	94.2	85.2	5.8	10.9	21.3
**0.25fmol**	**DAPAR**	135	3466	10718	2	98.5	75.6	3.7	7.2	16.6
	**MI4P**	133	1974	12210	4	97.1	86.1	6.3	11.9	22.9
**0.5fmol**	**DAPAR**	134	2495	11689	3	97.8	82.4	5.1	9.7	20.2
	**MI4P**	132	1233	12951	5	96.4	91.3	9.7	17.6	29.1
**1.25fmol**	**DAPAR**	132	2118	12066	5	96.4	85.1	5.9	11.1	21.8
	**MI4P**	129	792	13392	8	94.2	94.4	14	24.4	35.1
**2.5fmol**	**DAPAR**	125	473	13711	12	91.2	96.7	20.9	34	42.8
	**MI4P**	93	145	14039	44	67.9	99	39.1	49.6	50.9
**5fmol**	**DAPAR**	122	1100	13084	15	89.1	92.2	10	18	28.3
	**MI4P**	85	383	13801	52	62	97.3	18.2	28.1	32.5

S19 Table. Performance evaluation on the *Arabidopsis thaliana* + UPS1 dataset, filtered with at least 1 quantified value in each condition. Missing values were imputed using the maximum likelihood estimation method.
