# Supplementary material for: Accounting for multiple imputation-induced variability for differential analysis in mass spectrometry-based label-free quantitative proteomics
Source: PLoS Comput Biol. 2022 Aug 29;18(8):e1010420. doi: 10.1371/journal.pcbi.1010420 (PMC9462777; doi:10.1371/journal.pcbi.1010420)
Supplement: S20 Table — Missing values were imputed using the maximum likelihood estimation method. (PDF) [file pcbi.1010420.s020.pdf]

| Condition (vs 10fmol) | Method | True positives | False positives | True negatives | False negatives | Sensitivity (%) | Specificity (%) | Precision (%) | F-score (%) | MCC (%) |
|---|---|---|---|---|---|---|---|---|---|---|
| **5fmol** | **DAPAR** | 372 | 226 | 15522 | 196 | 65.5 | 98.6 | 62.2 | 63.8 | 62.5 |
| | **MI4P** | 348 | 179 | 15569 | 220 | 61.3 | 98.9 | 66 | 63.6 | 62.3 |

**S20 Table. Performance evaluation on the *Arabidopsis thaliana* + UPS1 dataset, filtered with at least 1 quantified value in each condition and focusing only on the comparison 5fmol vs. 10fmol.** Missing values were imputed using the maximum likelihood estimation method.
