# Supplementary material for: Accounting for multiple imputation-induced variability for differential analysis in mass spectrometry-based label-free quantitative proteomics
Source: PLoS Comput Biol. 2022 Aug 29;18(8):e1010420. doi: 10.1371/journal.pcbi.1010420 (PMC9462777; doi:10.1371/journal.pcbi.1010420)
Supplement: S21 Table — Missing values were imputed using the maximum likelihood estimation method. (PDF) [file pcbi.1010420.s021.pdf]

Condition (vs 10fmol)	Method	True positives	False positives	True negatives	False negatives	Sensitivity (%)	Specificity (%)	Precision (%)	F-score (%)	MCC (%)
**0.05fmol**	**DAPAR**	74	2989	8880	3	96.1	74.8	2.4	4.7	13
	**MI4P**	74	2989	8880	3	96.1	74.8	2.4	4.7	13
**0.25fmol**	**DAPAR**	76	2837	9032	1	98.7	76.1	2.6	5.1	13.9
	**MI4P**	76	2837	9032	1	98.7	76.1	2.6	5.1	13.9
**0.5fmol**	**DAPAR**	76	1905	9964	1	98.7	83.9	3.8	7.4	17.8
	**MI4P**	76	1905	9964	1	98.7	83.9	3.8	7.4	17.8
**1.25fmol**	**DAPAR**	75	1411	10458	2	97.4	88.1	5	9.6	20.7
	**MI4P**	75	1411	10458	2	97.4	88.1	5	9.6	20.7
**2.5fmol**	**DAPAR**	70	232	11637	7	90.9	98	23.2	36.9	45.3
	**MI4P**	70	232	11637	7	90.9	98	23.2	36.9	45.3
**5fmol**	**DAPAR**	67	686	11183	10	87	94.2	8.9	16.1	26.7
	**MI4P**	67	686	11183	10	87	94.2	8.9	16.1	26.7

S21 Table. Performance evaluation on the *Arabidopsis thaliana* + UPS1 dataset, filtered with at least 2 quantified values in each condition. Missing values were imputed using the maximum likelihood estimation method.
