# Supplementary material for: Accounting for multiple imputation-induced variability for differential analysis in mass spectrometry-based label-free quantitative proteomics
Source: PLoS Comput Biol. 2022 Aug 29;18(8):e1010420. doi: 10.1371/journal.pcbi.1010420 (PMC9462777; doi:10.1371/journal.pcbi.1010420)
Supplement: S22 Table — Missing values were imputed using the maximum likelihood estimation method. (PDF) [file pcbi.1010420.s022.pdf]

Condition (vs 10fmol)	Method	True positives	False positives	True negatives	False negatives	Sensitivity (%)	Specificity (%)	Precision (%)	F-score (%)	MCC (%)
**0.05fmol**	**DAPAR**	16	1567	6173	1	94.1	79.8	1	2	8.6
	**MI4P**	16	1567	6173	1	94.1	79.8	1	2	8.6
**0.25fmol**	**DAPAR**	16	1461	6279	1	94.1	81.1	1.1	2.1	9
	**MI4P**	16	1461	6279	1	94.1	81.1	1.1	2.1	9
**0.5fmol**	**DAPAR**	15	895	6845	2	88.2	88.4	1.6	3.2	11.1
	**MI4P**	15	895	6845	2	88.2	88.4	1.6	3.2	11.1
**1.25fmol**	**DAPAR**	16	880	6860	1	94.1	88.6	1.8	3.5	12.1
	**MI4P**	16	880	6860	1	94.1	88.6	1.8	3.5	12.1
**2.5fmol**	**DAPAR**	13	139	7601	4	76.5	98.2	8.6	15.4	25.2
	**MI4P**	13	139	7601	4	76.5	98.2	8.6	15.4	25.2
**5fmol**	**DAPAR**	11	419	7321	6	64.7	94.6	2.6	4.9	12.1
	**MI4P**	11	419	7321	6	64.7	94.6	2.6	4.9	12.1

S22 Table. Performance evaluation on the *Arabidopsis thaliana* + UPS1 dataset, extracted without Match Between Runs and filtered with at least 1 quantified value in each condition. Missing values were imputed using the maximum likelihood estimation method.
