# Supplementary material for: Accounting for multiple imputation-induced variability for differential analysis in mass spectrometry-based label-free quantitative proteomics
Source: PLoS Comput Biol. 2022 Aug 29;18(8):e1010420. doi: 10.1371/journal.pcbi.1010420 (PMC9462777; doi:10.1371/journal.pcbi.1010420)
Supplement: S23 Table — Missing values were imputed using the maximum likelihood estimation method. (PDF) [file pcbi.1010420.s023.pdf]

Condition (vs 10fmol)	Method	True positives	False positives	True negatives	False negatives	Sensitivity (%)	Specificity (%)	Precision (%)	F-score (%)	MCC (%)
**0.05fmol**	**DAPAR**	8	1234	4119	1	88.9	76.9	0.6	1.3	6.4
	**MI4P**	8	1234	4119	1	88.9	76.9	0.6	1.3	6.4
**0.25fmol**	**DAPAR**	8	1150	4203	1	88.9	78.5	0.7	1.4	6.7
	**MI4P**	8	1150	4203	1	88.9	78.5	0.7	1.4	6.7
**0.5fmol**	**DAPAR**	8	742	4611	1	88.9	86.1	1.1	2.1	8.9
	**MI4P**	8	742	4611	1	88.9	86.1	1.1	2.1	8.9
**1.25fmol**	**DAPAR**	8	536	4817	1	88.9	90	1.5	2.9	10.7
	**MI4P**	8	536	4817	1	88.9	90	1.5	2.9	10.7
**2.5fmol**	**DAPAR**	6	83	5270	3	66.7	98.4	6.7	12.2	20.9
	**MI4P**	6	83	5270	3	66.7	98.4	6.7	12.2	20.9
**5fmol**	**DAPAR**	6	274	5079	3	66.7	94.9	2.1	4.2	11.3
	**MI4P**	6	274	5079	3	66.7	94.9	2.1	4.2	11.3

S23 Table. Performance evaluation on the *Arabidopsis thaliana* + UPS1 dataset, extracted without Match Between Runs and filtered with at least 2 quantified value in each condition. Missing values were imputed using the maximum likelihood estimation method.
