# Supplementary material for: Accounting for multiple imputation-induced variability for differential analysis in mass spectrometry-based label-free quantitative proteomics
Source: PLoS Comput Biol. 2022 Aug 29;18(8):e1010420. doi: 10.1371/journal.pcbi.1010420 (PMC9462777; doi:10.1371/journal.pcbi.1010420)
Supplement: S24 Table — Missing values were imputed using the maximum likelihood estimation method. (PDF) [file pcbi.1010420.s024.pdf]

Condition (vs 10fmol)	Method	True positives	False positives	True negatives	False negatives	Sensitivity (%)	Specificity (%)	Precision (%)	F-score (%)	MCC (%)
**0.05fmol**	**DAPAR**	41	1040	1557	0	100	60	3.8	7.3	15.1
	**MI4P**	41	753	1844	0	100	71	5.2	9.8	19.1
**0.25fmol**	**DAPAR**	41	1072	1525	0	100	58.7	3.7	7.1	14.7
	**MI4P**	41	797	1800	0	100	69.3	4.9	9.3	18.4
**0.5fmol**	**DAPAR**	40	848	1749	1	97.6	67.3	4.5	8.6	17
	**MI4P**	40	585	2012	1	97.6	77.5	6.4	12	21.8
**1.25fmol**	**DAPAR**	41	409	2188	0	100	84.3	9.1	16.7	27.7
	**MI4P**	41	142	2455	0	100	94.5	22.4	36.6	46
**2.5fmol**	**DAPAR**	41	208	2389	0	100	92	16.5	28.3	38.9
	**MI4P**	40	69	2528	1	97.6	97.3	36.7	53.3	59
**5fmol**	**DAPAR**	41	475	2122	0	100	81.7	7.9	14.7	25.5
	**MI4P**	37	203	2394	4	90.2	92.2	15.4	26.3	35.5

S24 Table. Performance evaluation on the *Arabidopsis thaliana* + UPS1 dataset at the protein-level, filtered with at least 1 quantified values in each condition. Missing values were imputed using the maximum likelihood estimation method.
