# Supplementary material for: Accounting for multiple imputation-induced variability for differential analysis in mass spectrometry-based label-free quantitative proteomics
Source: PLoS Comput Biol. 2022 Aug 29;18(8):e1010420. doi: 10.1371/journal.pcbi.1010420 (PMC9462777; doi:10.1371/journal.pcbi.1010420)
Supplement: S25 Table — Missing values were imputed using the maximum likelihood estimation method. (PDF) [file pcbi.1010420.s025.pdf]

Condition (vs 25fmol)	Method	True positives	False positives	True negatives	False negatives	Sensitivity (%)	Specificity (%)	Precision (%)	F-score (%)	MCC (%)
**0.5fmol**	**DAPAR**	188	439	18067	4	97.9	97.6	30	45.9	53.5
	**MI4P**	183	144	18362	9	95.3	99.2	56	70.5	72.7
**1fmol**	**DAPAR**	186	246	18260	6	96.9	98.7	43.1	59.6	64.1
	**MI4P**	183	71	18435	9	95.3	99.6	72	82.1	82.7
**2.5fmol**	**DAPAR**	185	161	18345	7	96.4	99.1	53.5	68.8	71.4
	**MI4P**	179	39	18467	13	93.2	99.8	82.1	87.3	87.4
**5fmol**	**DAPAR**	182	108	18398	10	94.8	99.4	62.8	75.5	76.9
	**MI4P**	156	23	18483	36	81.2	99.9	87.2	84.1	84
**10fmol**	**DAPAR**	148	109	18397	44	77.1	99.4	57.6	65.9	66.2
	**MI4P**	86	27	18479	106	44.8	99.9	76.1	56.4	58.1

S25 Table. Performance evaluation on the *Saccharomyces cerevisiae* + UPS1 dataset, filtered with at least 1 quantified value in each condition. Missing values were imputed using the maximum likelihood estimation method.
