# Supplementary material for: Accounting for multiple imputation-induced variability for differential analysis in mass spectrometry-based label-free quantitative proteomics
Source: PLoS Comput Biol. 2022 Aug 29;18(8):e1010420. doi: 10.1371/journal.pcbi.1010420 (PMC9462777; doi:10.1371/journal.pcbi.1010420)
Supplement: S26 Table — Missing values were imputed using the maximum likelihood estimation method. (PDF) [file pcbi.1010420.s026.pdf]

Condition (vs 25fmol)	Method	True positives	False positives	True negatives	False negatives	Sensitivity (%)	Specificity (%)	Precision (%)	F-score (%)	MCC (%)
**0.5fmol**	**DAPAR**	131	146	16316	4	97	99.1	47.3	63.6	67.4
	**MI4P**	131	146	16316	4	97	99.1	47.3	63.6	67.4
**1fmol**	**DAPAR**	130	59	16403	5	96.3	99.6	68.8	80.2	81.2
	**MI4P**	130	59	16403	5	96.3	99.6	68.8	80.2	81.2
**2.5fmol**	**DAPAR**	130	30	16432	5	96.3	99.8	81.2	88.1	88.4
	**MI4P**	130	30	16432	5	96.3	99.8	81.2	88.1	88.4
**5fmol**	**DAPAR**	127	19	16443	8	94.1	99.9	87	90.4	90.4
	**MI4P**	127	19	16443	8	94.1	99.9	87	90.4	90.4
**10fmol**	**DAPAR**	96	18	16444	39	71.1	99.9	84.2	77.1	77.2
	**MI4P**	96	18	16444	39	71.1	99.9	84.2	77.1	77.2

S26 Table. Performance evaluation on the *Saccharomyces cerevisiae* + UPS1 dataset, filtered with at least 2 quantified values in each condition. Missing values were imputed using the maximum likelihood estimation method.
