# Supplementary material for: Accounting for multiple imputation-induced variability for differential analysis in mass spectrometry-based label-free quantitative proteomics
Source: PLoS Comput Biol. 2022 Aug 29;18(8):e1010420. doi: 10.1371/journal.pcbi.1010420 (PMC9462777; doi:10.1371/journal.pcbi.1010420)
Supplement: S27 Table — Missing values were imputed using the maximum likelihood estimation method. (PDF) [file pcbi.1010420.s027.pdf]

Condition (vs 25fmol)	Method	True positives	False positives	True negatives	False negatives	Sensitivity (%)	Specificity (%)	Precision (%)	F-score (%)	MCC (%)
**0.5fmol**	**DAPAR**	42	90	2285	0	100	96.2	31.8	48.3	55.3
	**MI4P**	42	24	2351	0	100	99	63.6	77.8	79.4
**1fmol**	**DAPAR**	42	65	2310	0	100	97.3	39.3	56.4	61.8
	**MI4P**	41	13	2362	1	97.6	99.5	75.9	85.4	85.8
**2.5fmol**	**DAPAR**	41	27	2348	1	97.6	98.9	60.3	74.5	76.2
	**MI4P**	41	8	2367	1	97.6	99.7	83.7	90.1	90.2
**5fmol**	**DAPAR**	42	19	2356	0	100	99.2	68.9	81.6	82.6
	**MI4P**	41	7	2368	1	97.6	99.7	85.4	91.1	91.2
**10fmol**	**DAPAR**	39	23	2352	3	92.9	99	62.9	75	75.9
	**MI4P**	38	7	2368	4	90.5	99.7	84.4	87.4	87.2

S27 Table. Performance evaluation on the *Saccharomyces cerevisiae* + UPS1 dataset, at the protein-level and filtered with at least 1 quantified values in each condition. Missing values were imputed using the maximum likelihood estimation method.
