# Supplementary material for: Accounting for multiple imputation-induced variability for differential analysis in mass spectrometry-based label-free quantitative proteomics
Source: PLoS Comput Biol. 2022 Aug 29;18(8):e1010420. doi: 10.1371/journal.pcbi.1010420 (PMC9462777; doi:10.1371/journal.pcbi.1010420)
Supplement: Attachment — Submitted filename: review_Plos_Comp_biol.pdf [file pcbi.1010420.s028.pdf]

This article by Chion *et al.* describes mi4p, a new R package for multiple imputation in mass spectrometry (MS)-based proteomics. Even the most modern proteomic datasets contain plenty of missing values because (1) peptide ions are absent or fall below the limit of detection (hence no MS1 peak above background noise is present), (2) MS1 peaks were not targeted for fragmentation (in case of data-dependent acquisition; hence no MS2 spectrum is present to identify the MS1 peak), or (3) cannot be (unambiguously) identified based on their MS2 spectra.

Most data analysis workflows revert to a single imputation approach to replace missing values with substitute values. In the downstream analysis, these imputed values are treated the same as observed values, which ignores the substantial uncertainty created by the imputation and leads to biased estimations for a substantial part of the proteome (i.e. every protein that contains missing values).

Multiple imputation tries to address this issue by imputing the dataset multiple times with different values and subsequently calculating the variability caused by the imputation procedure. In this work, the authors calculate for each peptide with missing values a variance-covariance matrix based on multiple imputed datasets. When inferring differential abundance, the standard deviation for each peptide with imputed values is then multiplied by the maximal diagonal element of the variance-covariance matrix from the imputation step. This procedure thus accounts for the additional uncertainty caused by the imputation. The authors prove that the theoretical superiority of their method compared to single imputation translates both to simulated datasets and to experimental spike-in datasets.

The superiority of multiple imputation as compared to single imputation has already long been established in the statistical community. However, multiple imputation has never really been adopted in the field of proteomics. This slow adoption of multiple imputation in the field is likely due to the absence of user-friendly software tailored towards proteomics data analysis. Therefore, the mi4p R package is innovative and addresses a real need for the proteomics community. The science in this article is solid, and the article is well written. I therefore recommend publication in PLOS Computation Biology, after the following comments are addressed.

Major comments

1. It is not entirely clear to me which methods were used to impute in the results section. If I explore the “multi.impute” function of the mi4p package, there are many imputation methods to choose from, but it is not clearly indicated in the manuscript which method was used. Similarly, it should be specified which single imputation method was used in the DAPAR package (both in the manuscript and in the figures). In the R Documentation of the package itself, I would also add some descriptions (or refer to the descriptions in other packages) as well as literature references for the imputation methods (e.g. "pmm", “midastouch”, etc.) that can be chosen from.

Minor comments

- **Line 34:** The authors write “Either the considered peptide is missing in the given biological sample, and the intensity is then missing not at random (MNAR), or it was missed during the acquisition process, and the intensity is then missing at random (MAR).” I think that missingness during the acquisition process can also be MNAR, e.g. lowly abundant peptides are much less likely to be targeted for fragmentation in DDA, and are hence more likely to be missed during the acquisition process. I would be a bit more specific; I think the given example of true absence (or a peptide being below the detection limit) is a good example of MNAR; examples of MAR could be e.g.

failed identification due to a biochemical modification that was not searched for or co-elution with another peptide, preventing accurate identification of the peptide.

- **Line 46:** The authors write “Recently, a single imputation method based on a hurdle model was implemented in the MSqRob R package.”. This statement is not completely correct. The hurdle model does not make use of imputation, it rather handles missing peptides as zero counts when doing statistical inference. It consists of a binary component that models whether a peptide is detected or not, and a normal component (MSqRob) that models the $\log_2$ intensities of the peptides that passed the detection hurdle. I would rather e.g. write: “The authors of the MSqRob R package recently proposed to bypass imputation with a hurdle model that combines count-based differential detection with intensity-based differential abundance.” Or if it needs to be shorter e.g.: “The authors of the MSqRob R package recently proposed a hurdle model to bypass imputation altogether.”, or something along those lines.
- **Line Fig. 6:** MCAR is only introduced in Fig. 6, but the abbreviation is not explained in the text. I would suggest mentioning “MCAR” together with “MAR” and “MNAR” in the introduction.
- On the readme page of the mi4p GitHub portal (https://github.com/mariechion/mi4p), some output is very long. I would suggest to only print out a limited number of rows by using an R function like “head” or the “[” function.
