# Supplementary material for: Accounting for multiple imputation-induced variability for differential analysis in mass spectrometry-based label-free quantitative proteomics
Source: PLoS Comput Biol. 2022 Aug 29;18(8):e1010420. doi: 10.1371/journal.pcbi.1010420 (PMC9462777; doi:10.1371/journal.pcbi.1010420)
Supplement: Attachment — Submitted filename: response-to-reviewers.pdf [file pcbi.1010420.s030.pdf]

Accounting for multiple imputation-induced variability for differential analysis in mass spectrometry-based label-free quantitative proteomics

Marie Chion, Christine Carapito and Frédéric Bertrand

Response to reviewers

We wish to express our appreciation to the reviewers for the insightful comments, which have helped us to significantly improve our manuscript and to address the audience in a clearer way. According to the suggestions, we have thoroughly revised our manuscript. Please find the updated version enclosed. In the following, we want to respond to the comments made by the reviewers point by point. We have colored the changes in the track-change manuscript in blue (provided as an additional file) and added the specific question number in front to make it easier for the reviewers.

Reviewer 1

1.1. *It is not entirely clear to me which methods were used to impute in the results section. If I explore the "multi.impute" function of the mi4p package, there are many imputation methods to choose from, but it is not clearly indicated in the manuscript which method was used. Similarly, it should be specified which single imputation method was used in the DAPAR package (both in the manuscript and in the figures). In the R Documentation of the package itself, I would also add some descriptions (or refer to the descriptions in other packages) as well as literature references for the imputation methods (e.g. "pmm", "midastouch", etc.) that can be chosen from.*

The results detailed in the "Results and discussion" section refer to the use of maximum likelihood estimation (MLE) method for imputing missing values. The same imputed datasets were used in the mi4p package and in the DAPAR package. Hence, only the combination method of the imputed datasets and its impact on the testing step are compared. A figure (Figure 1 in the "Indicators of performance" subsection from the "Methods" section) was added as an illustration of the comparison framework. The use of the MLE method was also precised in the captions of Figures 8, 9, 10 and 11 in the "Results and discussion" section. As far as the mi4p package is concerned, the documentation for the "multi.impute" function was updated and references to the descriptions in other packages were added. In addition, a subsection dedicated to the comparison of imputation methods was added in the "Results and discussion" section (see comment 3.4).

1.2. *Line 34: The authors write "Either the considered peptide is missing in the given biological sample, and the intensity is then missing not at random (MNAR), or it was missed during the acquisition process, and the intensity is then missing at random (MAR)." I think that missingness during the acquisition process can also be MNAR, e.g. lowly abundant peptides are much less likely to be targeted for fragmentation in DDA, and are hence more likely to be missed during the acquisition process. I would be a bit more specific; I think the given example of true absence (or a peptide being below the detection limit) is a good example of MNAR; examples of MAR could be e.g. failed identification due to a biochemical modification that was not searched for or co-elution with another peptide, preventing accurate identification of the peptide.*

We accounted for the recommended examples and rewrote the paragraph as follows:

"Missing peptides' intensities arise from various reasons (biological, analytical, bioinformatical) and obey different missing values mechanisms. For example, the considered peptide is missing in the given biological sample, and the intensity is then missing not at random (MNAR) or it could have not been accurately identified (non searched biochemical modification or peptides co-elution, ...), and the intensity is then missing at random (MAR)."

1.3. *Line 46: The authors write "Recently, a single imputation method based on a hurdle model was implemented in the MSqRob R package.". This statement is not completely correct. The hurdle model does not make use of imputation, it rather handles missing peptides as zero counts when doing statistical inference. It consists of a binary component that models whether a peptide is detected or not, and a normal component (MSqRob) that models the log2 intensities of the peptides that passed the detection hurdle. I would rather e.g. write: "The authors of the MSqRob R package recently proposed to bypass imputation with a hurdle model that combines count-based differential detection with intensity-based differential abundance." Or if it needs to be shorter e.g.: "The authors of the MSqRob R package recently proposed a hurdle model to bypass imputation altogether.", or something along those lines.*

The statement about the hurdle model was corrected according to the reviewer's suggestion as follows:

"Note that the authors of the MSqRob R package recently proposed to bypass imputation with a hurdle model that combines count-based differential detection with intensity-based differential abundance [9]."

1.4. *Line Fig. 6: MCAR is only introduced in Fig. 6, but the abbreviation is not explained in the text. I would suggest mentioning "MCAR" together with "MAR" and "MNAR" in the introduction.*

We added the explanation for the MCAR abbreviation when first mentioned in the "Simulated datasets under Missing Completely At Random and Not At Random assumption" subsection of the "Results and discussion" section:

"The previous results were provided using only missing at random data. This section extends the simulation study to a mixture of missing completely at random (MCAR) and missing not at random (MNAR) data. "

1.5. *On the readme page of the mi4p GitHub portal, some output is very long. I would suggest to only print out a limited number of rows by using an R function like "head" or the "[" function.*

The long outputs were indeed inconvenient. We have shortened the outputs on the readme page.

Reviewer 2

We thank the reviewer for his positive feedback.

Reviewer 3

3.1. *In the Author Summary, the authors state "Some state-of-the-art statistical proteomics data processing software proposes to impute these missing values, while others simply remove proteins with too many missing peptides." This discounts methods that combine quantitative and qualitative statistical methods. Later in the Introduction the authors state "Another way to cope with missing data is to use methods that account for the missing information" which alludes to the aforementioned methods. Direct citations to these methods (e.g. Webb-Robertson et al (2010). Combined statistical analyses of peptide intensities and peptide occurrences improves identification of significant peptides from MS-based proteomics data. Journal of proteome research) should be made. And the authors skip over this option straight to imputation without justification. Unless a justification can be provided as to why it's not relevant, the combined testing method is a perfectly viable option that should be compared to sensitivity, specificity, etc. of results based on imputation methods.*

Mention to methods that combine quantitative and qualitative methods as in Webb-Robertson et al. (2010) was added to the introduction. Our work focuses on the use of imputation methods to deal with missing values. Hence, in our real data analyses, we discarded peptides that had "too many" missing values by filtering them out (at least 1 or 2 quantified values in each condition). The approach suggested by Webb-Robertson et al. (2010) includes a G-test step that may be combined with our methodology. For instance, the G-test, corrected for multiple tests, can be applied before or after filtering out those peptides to search for peptides featuring significant differences in absence/presence throughout the conditions. From a general point of view this looks like a good idea. Yet, we will now give some insights on why a combined testing method is not always a perfectly viable option as it is not relevant, for instance, for the real datasets that we analyzed. We applied the G-test to the Arabidopsis real dataset, before filtering and it lead to 488 significant, at the .05 level, peptides with a Benjamini-Hochberg correction for multiple tests (2212 significant peptides for raw p-values). Hence, one may think that the G-test is

complementary to our approach and allows to spot relevant peptides that we filtered out by applying a classical experimental pretreatment. Note that only 84 peptides were found significant by raw G-test after filtering and 0 by BH corrected G-test after filtering. Hence most of the peptides found significant by the G-tests can not be found significant by our methodology.

Yet, both human and arabidopsis peptides can be found in our experimental datasets. Human peptides were spiked in the samples in varying amounts and are thus to be detected as varying significantly between two conditions. On the contrary, arabidopsis peptides are used as a constant background and hence are not to be detected as varying significantly between two conditions. Among the 488 peptides detected as significant by the G-test with BH correction, 280 are arabidopsis while only 208 are human peptides, a ratio of 280/208=1.35. For raw G-test p-values, it is even worse as 1767 arabidopsis peptides and 445 human peptides were found significant by the raw G-test, leading to a ratio of 1767/445=3.97. As a result, a hybrid method using the G-test will decrease the precision of our approach since most of the peptides found significant by the G-tests can not be found significant by our methodology. There is thus a need to adapt the G-test for specific experimental designs. The same phenomenon occurs if the G-test is applied after the filtering step. This is one of the reasons why we did not consider this type of presence/absence test for our datasets.

We nevertheless added a reference to the G-test in the readme of the package since it may be appropriate for other experimental designs.

3.2. *In the Introduction (line 43), the authors statement "The Perseus software [6] offers three methods for single imputation: either imputing by "NaN" ..." does not make sense. Does this mean missing values are replaced by NAs and are ignored in downstream analyses?*

In the Perseus software and according to its documentation, missing values can indeed be imputed by "NaN" and deleted from the quantitative matrix. Hence, missing values are ignored in downstream analyses. The precision has been added to the manuscript as follows:

"The Perseus software [7] offers three methods for single imputation: either imputing by "NaN" (hence ignoring missing values in downstream analysis), impute by a user-defined constant or impute according to a Gaussian distribution in order to simulate intensities, which are lower than the limit of detection."

See Perseus documentation: `http://www.coxdocs.org/doku.php?id=perseus:user:activities:matrixprocessing:imputation:replaceimputedbynan`

3.3. *On line 50 the authors state "The ProStaR software based on the DAPAR R package and developed by [9] splits missing values into two categories, whether they are Missing in an Entire Condition (MEC) or Partially Observed Values (POV)" This seems to be superfluous information, as following that statement, no details are given about why these matter or if the two groups are treated differently in imputation.*

In the DAPAR R package the user can indeed choose to impute the missing values according to their nature, whether they are MEC or POV. We added this detail to our statement as follows in the manuscript:

"The ProStaR software based on the DAPAR R package splits missing values into two categories, whether they are Missing in an Entire Condition (MEC) or Partially Observed Values (POV) and allow them to be imputed using different methods [10,11]."

3.4. *The choice of imputation algorithms is weakly justified, and very little attention is given to whether there are real differences between the results for different methods. The discussion and justification need to be bolstered, if all methods remain in the manuscript. I believe that more attention and discussion around the points in #8 (3.8.) would be of far more interest to the research community.*

The subset of the imputation methods (described in Table 1) was chosen according to the common imputation methods used in the recent articles on imputation in proteomics (described in S1 Table). However, the "Results and discussion" section of the manuscript provides results using the maximum likelihood estimation method (MLE) for missing values imputation.

We added a whole subsection on the comparison of the imputation methods from our subset, which explains this choice of focus, as described hereafter. Results for other imputation methods are available in the Supplementary Information.

Comparison of imputation methodologies

To compare the imputation methods considered in Table 1, we used the synthetic data from the aforementioned second set of MAR simulations. Let us highlight that reviews on imputation methods evaluation often base their study on real datasets by subsetting them to complete data and amputating them afterwards (S1 Table). However, such approaches remain limited, as the parameters of the data cannot be controlled. Recall that we simulated 100 datasets, which were amputated afterwards. Hence both imputed and real values can be accessed. In this section, we aim at evaluating the potential bias that can arise from the imputation process. We based our comparison on the amputated datasets with a proportion of missing values of 10%, so we impute each dataset $D_Q = 10$ times. Consider then the set of all missing values coming from the $Q = 100$ datasets. Let n_q denote the number of missing values in the q-th dataset, with $1 \leq q \leq Q$. The set of all missing data is then constituted of $N_Q = \sum_{q=1}^{Q} n_q$ elements. In our work, we take the number of draws for multiple imputation as the percentage of missing values. Therefore, multiple imputation produces ten vectors of size N_Q corresponding to the ten draws of the considered vector.

Imputation error for each draw To evaluate the performance of the imputation methodologies considered, we first consider the error on each draw. Let y_i denote the i-th value in the previously defined set and $y_i^{(d)}$ the d-th draw for y_i. Hence, we define the error $\varepsilon_i^{(d)}$ for each imputed value $y_i^{(d)}$ as:

$$\varepsilon_i^{(d)} = y_i^{(d)} - y_i, \forall i \in 1, \ldots, N_Q, \forall d \in 1, \ldots, D_Q.$$

The $D_Q \times N_Q$ errors are calculated for all imputation method considered, namely `kNN`, `MLE`, `norm`, `PCA` and `RF` (detailed in Table 1). To compare the performances of these methods, Fig 4 summarises the

Figure 4: **Distribution of empirical errors for the five imputation methods considered on the second set of MAR simulations.**

distributions of $(\varepsilon_i^{(d)})_{i=1,\ldots,N_q,d=1,\ldots,D_Q}$ for the five imputation methods considered. First, it is comforting to observe that the errors are all centred on zero. Moreover, let us also point out that the `MLE` and `norm` methods provide a slightly increased variability than other methods. The `kNN`, `PCA` and `RF` methods show equivalent performance as far as single imputation is concerned.

Imputation error for the mean of draws Following the first Rubin's rule (Eq 1), the D_Q drawn datasets are combined using the mean. In order to provide additional insights about the empirical errors of the different multiple-imputation procedures, let us compute the differences between the averaged imputed values used in practice and the actual values. For each imputation method, the errors are averaged over the D_Q draws (corresponding to the D_Q different imputations), which we expect to stabilise the error values. In contrast to the previous approach, the associated formula becomes:

$$\varepsilon_i = \frac{1}{D_Q} \sum_{d=1}^{D_Q} y_i^{(d)} - y_i, \ \forall i \in 1, \ldots, N_Q.$$

Fig 5 suggests equivalent performance for all five methods as far as the mean of all imputed datasets is concerned. In terms of variability, we can still observe a slightly increased interquartile range for the `MLE` imputation method.

Figure 5: **Distribution of errors of the averaged imputed values for the five imputation methods considered on the second set of MAR simulations.**

Computation time As a complement to determine the advantages of each approach, we compared the running time of all imputation processes are compared as well. Therefore, we considered the total time needed for imputing D_Q times each simulated dataset. According to the boxplots on Fig 6 highlight the `MLE` and `kNN` method to be the fastest.Compared to `MLE` imputation method, the `PCA` method is on average 3.5 times slower and the `norm` and `RF` methods are respectively on average 7.4 times and 8.1 times slower. At this stage of the comparison, as all imputation methods exhibit comparable performances in terms of imputation bias, a preference can be drawn for the `kNN` and `MLE` methods.

Figure 6: **Distributions of duration of the imputation process for the five imputation methods considered on the second set of MAR simulations.**

Influence on testing results The evaluation of performance for our `mi4p` methodology relies on the results produced by the testing procedure. For the MAR simulation designs, testing results were provided for all imputation methods considered. However, we could observe that no positives were produced for some datasets. As a summary, Table 1 describes under which conditions such pathological cases arise in the second set of MAR simulations. The `mi4p` workflow dramatically underperforms at detecting positives when using the `norm` imputation method. The high number of pathological cases can be explained by this method being a global one (*i.e.* to the full dataset), whereas other methods considered are local in that they are applied experimental condition-wise. Therefore, the `norm` method might lead to an increased between-imputation variability. Otherwise, no pathological cases occur while using the `mi4p` method on this particular set of simulated datasets. However, a few pathological cases can be consistently observed when using the `DAPAR` workflow, regardless of the chosen imputation method. Overall, the `MLE` imputation offers a slight advantage over other methods.

Table 1: **Number of pathological cases for each simulation condition on the second set of MAR simulations.**

**Imputation method**	**Testing workflow**	**1%**	**5%**	**10%**	**15%**	**20%**	**25%**
**kNN**	**DAPAR**	0	0	2	2	2	1
	**MI4P**	0	0	0	0	0	0
**MLE**	**DAPAR**	0	0	2	1	1	0
	**MI4P**	0	0	0	0	0	0
**norm**	**DAPAR**	0	0	2	2	1	0
	**MI4P**	0	0	0	7	26	57
**PCA**	**DAPAR**	0	0	2	2	3	0
	**MI4P**	0	0	0	0	0	0
**RF**	**DAPAR**	0	0	3	2	3	0
	**MI4P**	0	0	0	0	0	0

A glimpse of real datasets imputation As a conclusion of this thorough analysis of synthetic data, let us draw some perspectives for the subsequent real datasets study. At this stage, `kNN` and `MLE` imputation methods might equivalently be considered. However, in quantitative proteomics datasets, rows sometimes present more than 50% missing values. When this threshold is exceeded, current kNN method implementations only use mean imputation for these rows. However, mean imputation results in identical imputed values and no between-imputation variability arises, preventing from taking advantage of our `mi4p` methodology.

In contrast, the `MLE` imputation method still provides reliable imputations for a reduced computational cost in all situations. Moreover, the `MLE` method offers a more principled and interpretable approach compared to alternatives, which also motivated our choice to retain this method for further analysis of both MNAR + MCAR simulated datasets and real datasets.

3.5. *There appears to be a lack of knowledge about the state-of-the-art with regard to proteomics data and imputation. Throughout the paper the authors mention that the imputation methods can be used at the peptide or protein level. ref. 31 demonstrated that missing value imputation is more efficient on peptides than on proteins. Unless the authors have evidence to the contrary, they should not be suggesting imputation at the protein level.*

We provide a methodology that can be applied to peptide-level as well as protein-level quantitative data, leaving the choice to the user. However, in the "Aggregation" subsection of the "Methods" section, we indeed suggest imputing at the peptide level, before inferring results at the protein level. As our statement seems to be unclear, we added the reference to Lazar et al. (2016) recommending peptide-level imputation.

"The methodology implemented in the `mi4p` `R` package can be applied to peptide-level quantification data as well as protein-level quantification data. However, common practice in proteomics consists in inferring results at the protein level from peptide-level data. In particular, imputation should be performed at the peptide level, before aggregating peptides into proteins [31]. Therefore, we adjusted our pipeline as follows: "

3.6. *On line 96 "We set the number of draws D equal to the proportion of missing values in the dataset" This statement does not make any sense. How do you draw e.g. 0.4 datasets?*

This statement is indeed confusing. We choose the ceiling of the proportion of missing values so if we would have 6.4% missing values, we would set the number of draws to 7. However, if the proportion of missing values is less than 1%, we set the number of draws to 2. This precision was added to the manuscript.

"We set the number of draws D equal to the ceiling of the proportion of missing values in the dataset [3]. Note that if the proportion of missing values is less than 1%, the number of draws is set to 2."

3.7. *There are some undefined parameters such as d_p and β_p. The authors should make it clear as to what the betas are representing here as well, non-stats savvy folks will not make the leap.*

We corrected this omission and added the description of d_p and β_p:

"The objective of multiple imputation is to estimate from D drawn datasets the vector of parameters of interest $\boldsymbol{\beta_p} = (\beta_{p1}, \ldots, \beta_{pK})$ (*e.g.* being the vector of coefficients of the linear model for peptide p) and its variance-covariance matrix Σ_p. "

"where σ_p^2 is the peptide-wise variance, d_p is the residual degrees of freedom for the linear model for peptide p, d_0 and s_0 are hyperparameters to be estimated [16]. "

"with $(\boldsymbol{X}^{\mathrm{T}}\boldsymbol{X})^{-1}_{j,j}$ the j-th diagonal element in the matrix $(\boldsymbol{X}^{\mathrm{T}}\boldsymbol{X})^{-1}$ and $\hat{\beta}_{pj}$ is the j-th coefficient of the linear model pour peptide p."

3.8. *The exercise to simulate datasets where missingness is due only to the Missing Completely at Random mechanism provides no value to the manuscript, as this does not occur in practice with real LC-MS datasets. Further, parameter choices in the hybrid missingness simulation lack justification. Do the % missingness match typical shotgun proteomics data (e.g. 1% doesn't seem plausible)? A log2 fold-change of 12.5, which equates to a fold-change of 5,792 does not seem within the realm of reasonableness at all "the 200 differentially expressed analytes have log-intensities drawn from a Gaussian distribution with a mean of 12.5 in the first condition and 25 in the second one". Differing number of samples/replicates should be considered at some level as well. For data that are not MCAR, differencing levels of abundance for different peptides should be simulated, and the simulations should be done such that the level of missingness is at least loosely tied to the overall abundance of each peptide, as is the case with real proteomics data.*

We provided a simulation studying relying on the one hand on the Missing At Random mechanism and on the other hand on a mixture of Missing Completely At Random and Missing Not At Random mechanisms. We considered various experimental designs as well as sample sizes. No simulated dataset is ideal, however, they are general practice in the field as they allow to challenge the methodology. Therefore, we mimicked what has been proposed by Karpievitch et al. (2012), Lazar et al. (2016) and Giai-Gianetto et al. (2020). In addition, we have used the simulated datasets only in a first instance before applying our method to a real, while controlled dataset. This framework offers a good trade-off between simulated data that can indeed be far from the reality and real data where some experimental parameters can not be controlled.

3.9. *How does this work translate to cases where there are more than two phenotypes to compare, and a t-test is no longer appropriate or the case where data are paired?*

The implementation of the aforementioned testing framework strongly relies on the `limma` `R` package. Hence, this work can be generalized to any design matrix. This precision was added to the "Hypotheses testing" subsection of the "Methods" section:

"Note that the implementation of the aforementioned testing framework strongly relies on the `limma` `R` package. Hence, this work can be generalised to any experimental design."

3.10. *Simulated data and/or code for simulation and analysis code should be made available as per the journal's policy.*

Simulated data and code were made available on Github: `https://github.com/mariechion/mi4p/` The functions used were implemented in a `R` package, called mi4p and available on the CRAN: `https://cran.r-project.org/package=mi4p`

The spiked yeast dataset is public and accessible on the ProteomeXchange website using the following identifier PXD003841. The reviewers can access the Arabidopsis thaliana spiked dataset using following identifiers: Username: `reviewer_pxd027800@ebi.ac.uk` Password: iBPyaetC

This dataset will be made publicly available at publication of the present manuscript.
