# Supplementary material for: Accounting for multiple imputation-induced variability for differential analysis in mass spectrometry-based label-free quantitative proteomics
Source: PLoS Comput Biol. 2022 Aug 29;18(8):e1010420. doi: 10.1371/journal.pcbi.1010420 (PMC9462777; doi:10.1371/journal.pcbi.1010420)
Supplement: Attachment — Submitted filename: response-to-reviewers_v2.pdf [file pcbi.1010420.s031.pdf]

Accounting for multiple imputation-induced variability for differential analysis in mass spectrometry-based label-free quantitative proteomics

Marie Chion, Christine Carapito and Frédéric Bertrand

Response to reviewers - Round 2

We wish to thank the reviewers on the positive feedback on our revised manuscript. We accounted for the suggestions for clarification. Please find the updated version enclosed. As previously, we have colored the changes in the track-change manuscript in blue (provided as an additional file) and responded to the comments point by point in the following.

Reviewer 1

Comment 1. *I am not sure what is meant by "pathological cases" in table 2 and the text preceding it. Do the authors mean the number of "false positives"? If so, they should use this terminology. If this interpretation is correct, I wonder why the authors picked this metric and not e.g. sensitivity, specificity or F-score for example? (this could be addressed by adding a sentence explaining why the authors compare these methods in terms of the numbers of false positives)*

In this context, a "pathological case" is a dataset for which no positive is found by the differential analysis. This means that for such a dataset, the moderated t-test provides no significantly differential peptide. In the simulation study, results are provided on simulated datasets using the mean ± standard deviation of the indicators of performance over the 100 simulated datasets (see Tables S2 to S18). As the number of pathological cases is low, the effect cannot be properly seen once the indicators are averaged over the 100 datasets. This is why we prefered to explicitly report the number of pathological cases in Table 2.

We replaced "pathological cases" by "pathological datasets" for the sake of clarity:

"As a summary, Table 2 describes under which conditions such pathological ~~cases~~ datasets arise in the second set of MAR simulations. (...) The high number of pathological ~~cases~~ datasets can be explained by this method being a global one (*i.e.* applied to the full dataset), whereas other methods considered are local in that they are applied experimental condition-wise. (...) However, a few pathological ~~cases~~ datasets can be consistently observed when using the `DAPAR` workflow, regardless of the chosen imputation method. "

We also added the precision on the mean ± standard deviation in the captions of Tables S2 to S18 of the Supporting Information, as such:

S2 Table. Performance evaluation on the first set of MAR simulations imputed using maximum likelihood estimation. Results are provided as mean ± standard deviation over the 100 simulated datasets for each indicator of performance.

S3 Table. Performance evaluation on the first set of MAR simulations imputed using k-nearest neighbours. Results are provided as mean ± standard deviation over the 100 simulated datasets for each indicator of performance.

S4 Table. Performance evaluation on the first set of MAR simulations imputed using Bayesian linear regression. Results are provided as mean ± standard deviation over the 100 simulated datasets for each indicator of performance.

S5 Table. Performance evaluation on the first set of MAR simulations imputed using principal component analysis. Results are provided as mean ± standard deviation over the 100 simulated datasets for each indicator of performance.

S6 Table. Performance evaluation on the first set of MAR simulations imputed using random forests. Results are provided as mean ± standard deviation over the 100 simulated datasets for each indicator of performance.

S7 Table. Performance evaluation on the second set of MAR simulations imputed using maximum likelihood estimation. Results are provided as mean ± standard deviation over the 100 simulated datasets for each indicator of performance.

S8 Table. Performance evaluation on the second set of MAR simulations imputed using k-nearest neighbours. Results are provided as mean ± standard deviation over the 100 simulated datasets for each indicator of performance.

S9 Table. Performance evaluation on the second set of MAR simulations imputed using Bayesian linear regression. Results are provided as mean ± standard deviation over the 100 simulated datasets for each indicator of performance.

S10 Table. Performance evaluation on the second set of MAR simulations imputed using principal component analysis. Results are provided as mean ± standard deviation over the 100 simulated datasets for each indicator of performance.

S11 Table. Performance evaluation on the second set of MAR simulations imputed using random forests. Results are provided as mean ± standard deviation over the 100 simulated datasets for each indicator of performance.

S12 Table. Performance evaluation on the third set of MAR simulations imputed using maximum likelihood estimation. Results are provided as mean ± standard deviation over the 100 simulated datasets for each indicator of performance.

S13 Table. Performance evaluation on the third set of MAR simulations imputed using k-nearest neighbours. Results are provided as mean ± standard deviation over the 100 simulated datasets for each indicator of performance.

S14 Table. Performance evaluation on the third set of MAR simulations imputed using Bayesian linear regression. Results are provided as mean ± standard deviation over the 100 simulated datasets for each indicator of performance.

S15 Table. Performance evaluation on the third set of MAR simulations imputed using principal component analysis. Results are provided as mean ± standard deviation over the 100 simulated datasets for each indicator of performance.

S16 Table. Performance evaluation on the third set of MAR simulations imputed using random forests. Results are provided as mean ± standard deviation over the 100 simulated datasets for each indicator of performance.

S17 Table. Performance evaluation on the first set of MCAR + MNAR simulations imputed using maximum likelihood estimation. Results are provided as mean ± standard deviation over the 100 simulated datasets for each indicator of performance.

S18 Table. Performance evaluation on the second set of MCAR + MNAR simulations imputed using maximum likelihood estimation. Results are provided as mean ± standard deviation over the 100 simulated datasets for each indicator of performance.

Comment 2. *Line 275: the authors state "The mi4p workflow dramatically underperforms at detecting positives when using the norm imputation method." I assume the term "positives" refers to "true positives"? If this interpretation is correct, is there somewhere a table with the number of true positives to back up this claim? Alternatively, if this sentence refers to the high number of false positives, I would rephrase this sentence accordingly, e.g. by saying that the mi4p workflow detects a lot of false positives when using the norm imputation method.*

The term "positives" refers to peptides which were detected as differentially abundant by the testing procedure, whether they are true or false positives. Our statement supports the previous text on the pathological datasets for each imputation methods and each missing value proportion. As explained in the Comment 1 and shown in Table 2, when the norm imputation method is used, no differentially abundant peptide is found in 7 out of 100 datasets with a 15% missing values (MV) proportion, in 26 out of 100 datasets with a 20% MV proportion and in 57 out of 100 datasets with a 25% MV proportion.

Comment 3. *I would also suggest to explicitly add a field to table 2 to make it clear from the table itself that the given percentages are the amputation percentages.*

We added a "Missing value proportion" field to Table 2 and changed the caption.

Table 3: **Number of pathological cases for each ~~simulation condition on~~ missing value proportion in the second set of MAR simulations.**

Imputation method	Testing workflow	Missing value proportion					
		1%	5%	10%	15%	20%	25%
kNN	DAPAR	0	0	2	2	2	1
	MI4P	0	0	0	0	0	0
MLE	DAPAR	0	0	2	1	1	0
	MI4P	0	0	0	0	0	0
norm	DAPAR	0	0	2	2	1	0
	MI4P	0	0	0	7	26	57
PCA	DAPAR	0	0	2	2	3	0
	MI4P	0	0	0	0	0	0
RF	DAPAR	0	0	3	2	3	0
	MI4P	0	0	0	0	0	0

Comment 4. *I further have some small grammar suggestions: Line 105: "coefficient of the linear model pour peptide p" -> "coefficient of the linear model for peptide p" Line 264: "a slightly increased variability than other methods" -> "a slightly increased variability compared to other methods" Line 270: remove "are compared as well" Line 271: "for imputing DQ times each simulated dataset" -> "for imputing each simulated dataset DQ times" Line 272: remove "According to"*

The grammar suggestions and the typos were corrected.

Reviewer 3

We thank the reviewer for his positive feedback.
