# Supplementary material for: Accounting for multiple imputation-induced variability for differential analysis in mass spectrometry-based label-free quantitative proteomics
Source: PLoS Comput Biol. 2022 Aug 29;18(8):e1010420. doi: 10.1371/journal.pcbi.1010420 (PMC9462777; doi:10.1371/journal.pcbi.1010420)
Supplement: Attachment — Submitted filename: PLOSCB_Chion_Review.docx [file pcbi.1010420.s029.docx]

Chion et al. propose an approach to appropriately utilize multiple imputation applied to shotgun proteomics. This is an important topic, as multiple imputation methods are often employed, but the primary strength of these methods (the ability to estimate variability in the imputation method) almost always goes unused by practitioners. Although an important topic, I feel that the manuscript has several missed opportunities and places for improvement.

My major issues with the manuscript is that the need for imputation is not well-justified, the data simulations are not realistic, and the authors don’t mention or discuss extensibility to other basic statistical comparisons (e.g. I have three phenotypes for which I wish to conduct an ANOVA). I would be more convinced if the authors were looking at imputation with an eye for doing classification or machine learning, where missing values are not permissible. In this case, statistics can be done using both quantitative and qualitative information and no comparison is offered to this approach. Specific comments are as follows:

1) In the Author Summary, the authors state “Some state-of-the-art statistical proteomics data processing software proposes to impute these missing values, while others simply remove proteins with too many missing peptides.” This discounts methods that combine quantitative and qualitative statistical methods. Later in the Introduction the authors state “Another way to cope with missing data is to use methods that account for the missing information” which alludes to the aforementioned methods. Direct citations to these methods (e.g. Webb-Robertson et al (2010). Combined statistical analyses of peptide intensities and peptide occurrences improves identification of significant peptides from MS-based proteomics data. *Journal of proteome research*) should be made. And the authors skip over this option straight to imputation without justification. Unless a justification can be provided as to why it's not relevant, the combined testing method is a perfectly viable option that should be compared to sensitivity, specificity, etc. of results based on imputation methods.
2) In the Introduction (line 43), the authors statement “The Perseus software [6] offers three methods for single imputation: either imputing by ”NaN”…” does not make sense. Does this mean missing values are replaced by NAs and are ignored in downstream analyses?
3) On line 50 the authors state “The ProStaR software based on the DAPAR R package and developed by [9] splits 50 missing values into two categories, whether they are Missing in an Entire Condition (MEC) or Partially Observed Values (POV)” This seems to be superfluous information, as following that statement, no details are given about why these matter or if the two groups are treated differently in imputation.
4) The choice of imputation algorithms is weakly justified, and very little attention is given to whether there are real differences between the results for different methods. The discussion and justification need to be bolstered, if all methods remain in the manuscript. I believe that more attention and discussion around the points in #8 would be of far more interest to the research community.
5) There appears to be a lack of knowledge about the state-of-the-art with regard to proteomics data and imputation. Throughout the paper the authors mention that the imputation methods can be used at the peptide or protein level. ref. 31 demonstrated that missing value imputation is more efficient on peptides than on proteins. Unless the authors have evidence to the contrary, they should not be suggesting imputation at the protein level.
6) On line 96 “We set the number of draws D equal to the proportion of missing values in the dataset” This statement does not make any sense. How do you draw e.g. 0.4 datasets?
7) There are some undefined parameters such as d_p_. The authors should make it clear as to what the betas are representing here as well, non-stats savvy folks will not make the leap.
8) The exercise to simulate datasets where missingness is due only to the Missing Completely at Random mechanism provides no value to the manuscript, as this does not occur in practice with real LC-MS datasets. Further, parameter choices in the hybrid missingness simulation lack justification. Do the % missingness match typical shotgun proteomics data (e.g. 1% doesn’t seem plausible)? A log2 fold-change of 12.5, which equates to a fold-change of 5,792 does not seem within the realm of reasonableness at all 🡪 “the 200 differentially expressed analytes have log-intensities drawn from a Gaussian distribution with a mean of 12.5 in the first condition and 25 in the second one”. Differing number of samples/replicates should be considered at some level as well. For data that are not MCAR, differening levels of abundance for different peptides should be simulated, and the simulations should be done such that the level of missingness is at least loosely tied to the overall abundance of each peptide, as is the case with real proteomics data.
9) How does this work translate to cases where there are more than two phenotypes to compare, and a t-test is no longer appropriate or the case where data are paired?
10) Simulated data and/or code for simulation and analysis code should be made available as per the journal’s policy.
